# Facile Synthesis of 5-Aryl-*N*-(pyrazin-2-yl)thiophene-2-carboxamides via Suzuki Cross-Coupling Reactions, Their Electronic and Nonlinear Optical Properties through DFT Calculations

**DOI:** 10.3390/molecules26237309

**Published:** 2021-12-02

**Authors:** Gulraiz Ahmad, Nasir Rasool, Adeel Mubarik, Ameer Fawad Zahoor, Muhammad Ali Hashmi, Muhammad Zubair, Muhammad Bilal, Mohamed Hussien, Muhammad Saeed Akhtar, Sajjad Haider

**Affiliations:** 1 Department of Chemistry, Government College, University Faisalabad, Faisalabad 38000, Pakistan; gulchemist35@gmail.com (G.A.); adeelmubarik2@gmail.com (A.M.); fawad.zahoor@gmail.com (A.F.Z.); zubairmkn@gcuf.edu.pk (M.Z.); muhammadbilalgcuf@gmail.com (M.B.); 2 Department of Chemistry, University of Education Lahore, Attock Campus, Attock 43600, Pakistan; i4hashmi@hotmail.com; 3 Department of Chemistry, Faculty of Science, King Khalid University, P.O. Box 9004, Abha 61413, Saudi Arabia; ma2464013@gmail.com; 4 School of Chemical Engineering, Yeungnam University, Gyeongsan 38541, Korea; msakhtar@ynu.ac.kr; 5 Chemical Engineering Department, College of Engineering, King Saud University, P.O. Box 800, Riyadh 11421, Saudi Arabia

**Keywords:** pyrazine, thiophenecarboxamide, Suzuki coupling, FMO analysis, NLO properties, ^1^H NMR comparison

## Abstract

Synthesis of 5-aryl-*N*-(pyrazin-2-yl)thiophene-2-carboxamides (**4a**–**4n**) by a Suzuki cross-coupling reaction of 5-bromo-*N*-(pyrazin-2-yl)thiophene-2-carboxamide (**3**) with various aryl/heteroaryl boronic acids/pinacol esters was observed in this article. The intermediate compound **3** was prepared by condensation of pyrazin-2-amine (**1**) with 5-bromothiophene-2-carboxylic acid (**2**) mediated by TiCl_4_. The target pyrazine analogs (**4a**–**4n**) were confirmed by NMR and mass spectrometry. In DFT calculation of target molecules, several reactivity parameters like FMOs (E_HOMO_, E_LUMO_), HOMO–LUMO energy gap, electron affinity (A), ionization energy (I), electrophilicity index (ω), chemical softness (σ) and chemical hardness (η) were considered and discussed. Effect of various substituents was observed on values of the HOMO–LUMO energy gap and hyperpolarizability. The p-electronic delocalization extended over pyrazine, benzene and thiophene was examined in studying the NLO behavior. The chemical shifts of ^1^H NMR of all the synthesized compounds **4a**–**4n** were calculated and compared with the experimental values.

## 1. Introduction

Pyrazine derivatives are a vital group of heterocyclic compounds present in nature and have also been synthesized in laboratories since 1876 [1,2]. Pyrazines and their derivatives play a significant role as intermediates for pharmaceuticals, agricultural chemicals [1], etc. In particular, pyrazine derivatives display a large number of pharmaceutical activities: anticancer [3], diuretic [4], antidiabetic [5] and anti-inflammatory [6]. Various synthetic procedures have been implemented to synthesize these biologically active pyrazine derivatives [7,8,9]. Naturally, these are found in tobacco, roasted and nut-like flavors and also the taste and fragrance of many foodstuffs [10].

**Figure 1:**
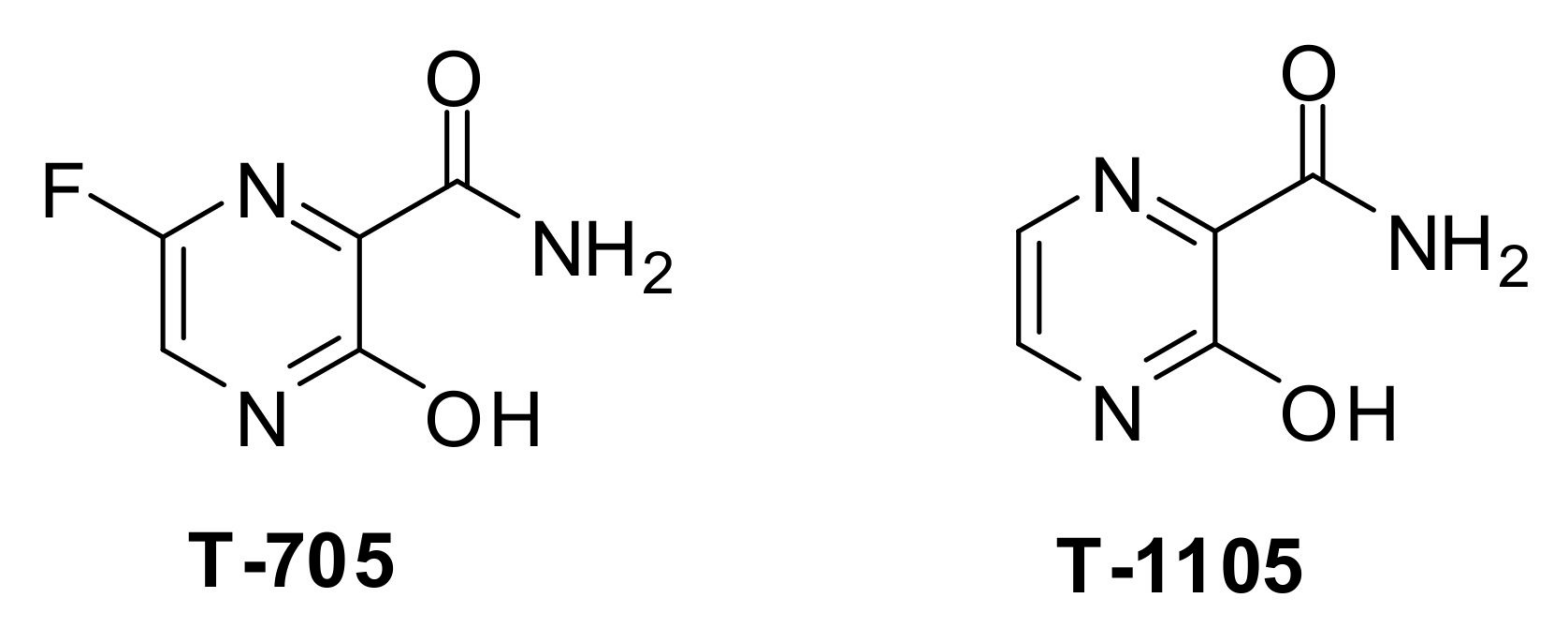
RNA viral polymerase inhibitors T-705 and T-1105.

Pyrazine carboxamide analogs like T-705 and T-1105 (Figure 1) are innovative broad-spectrum viral polymerase inhibitors against various RNA viruses [11,12,13]. T-705 is also studied in terms of treatment of several viral infections, including SARS-CoV-2 [14]. Pyrazinamide analogs were also studied for their high antimicrobial activity [15]. Pyrazinamide (PZA) is a vital first-line anti-tuberculosis agent and revealed a distinct inhibiting effect [16,17]. It contributes an exclusive role in shortening the therapy duration from more than nine months previously to six months as it kills semi-dormant tubercle bacilli in acidic environments compared to other TB drugs [18].

In recent years, organic NLO (nonlinear optical) materials have attained great interest due to their remarkable uses in photonics and optoelectronics comprising optical signal processing, optical communications, optical data storage and optoelectronic transfer [19,20,21]. To display prominent second-order NLO properties, a molecule should be non-centrosymmetric along with intramolecular charge transfer transitions, a large transition dipole moment and a huge difference in the molecular dipole moment at the ground and excited state [20,22]. It can be attained in linear organic compounds by substituting electron-donating and withdrawing groups as it happens in conventional organic dipolar push–pull systems [23].

Pyrazine compounds are a rigid planar conjugated structure, have electron-deficient character, and so the pyrazinyl moiety can be used as an electron-withdrawing group in electron push–pull systems [24]. Thus, pyrazine entities incorporation in luminescent materials has been studied recently [25]. Moreover, the electron push–pull pyrazine analogs exhibited fascinating second-order [26,27,28] and third-order [29,30,31] NLO properties. Therefore, in this manuscript, we elaborated on the production of a new sequence of 5-aryl-*N*-(pyrazin-2-yl)thiophene-2-carboxamides (**4a**–**4n**) and exploration of their NLO properties. We also focused on the structural reactivity parameters and ^1^H NMR spectra.

## 2. Results and Discussion

### 2.1. Chemistry

**Scheme 1:**
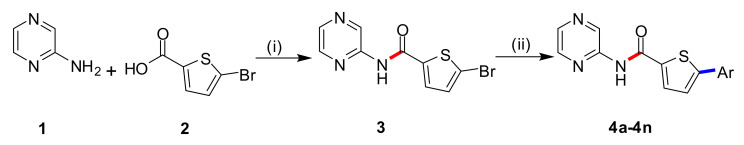
Synthesis and arylation of 5-bromo-*N*-(pyrazin-2-yl)thiophene-2-carboxamide (**3**) to form analogs (**4a**–**4n**).

**Figure 2:**
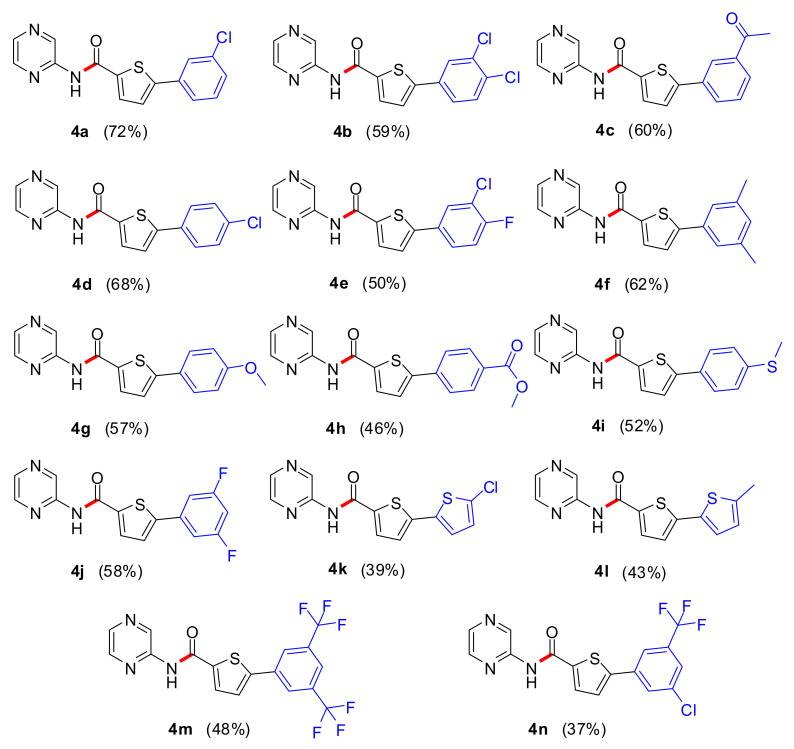
An overview of 5-bromo-*N*-(pyrazin-2-yl)thiophene-2-carboxamide derivatives via a Suzuki cross-coupling reaction.

The reaction of pyrazine-2-amine (**1**) with 5-bromothiophene-2-carboxylic acid (**2**) in the presence of pyridine and titanium tetrachloride was used to produce 5-bromo-*N*-(pyrazin-2-yl)thiophene-2-carboxamide (**3**) with 75% yield, as shown in Scheme 1. Thereafter, compound **3** was treated with different aryl boronic acids/pinacol esters by using catalyst Pd(PPh_3_)_4_, potassium phosphate and solvent 1,4-dioxane. The derivatives of 5-bromo-*N*-(pyrazin-2-yl)thiophene-2-carboxamide (**4a**–**4n**) were obtained with moderate and good yields (37–72%). The structural representation of these molecules and their yields are provided in Figure 2. Compound **4a** showed the maximum yield (72%), while compound **4n** exhibited the lowest yield (37%). The compounds having substitution 3,4-dichloro (**4b**), 3-methylcarbonyl (**4c**), 4-chloro (**4d**), 3-cholro4-fluoro (**4e**), 3,5-dimethyl (**4f**), 4-methoxy (**4g**), 4-methylthio (**4i**) and 3,5-difluoro (**4j**) were also synthesized with good yields. It was noted that the synthesized molecules obtained from thiophene boronic acid pinacol esters and bulky group substituted phenylboronic acid exhibited a low yield as compared to other molecules. It is noteworthy that the boronic acids and the pinacolate esters having electron-donating moieties provided good yields while the electron-withdrawing moieties provided lower yields [32]. Exceptionally, compound **4a** provided a good yield.

### 2.2. Computational Details

In this study, the geometric optimization of the synthesized molecules (**4a**–**4n**) followed by the computation of several quantum chemical parameters was studied through density functional theory (DFT) calculations. DFT is a cheap and widely used method for modeling the ground state of molecules. From the computational point of view, these methods have become very popular in recent years because they can attain similar accuracy to the ab initio methods in less time and at a lower computational cost [33]. The optimization of the molecules (**4a**–**4n**) was accomplished with the PBE0-D3BJ/def2-TZVP/SMD_1,4-dioxane_ level of theory [34] which is widely used for providing accurate geometries and electronic properties for a large number of molecules. The solvent model density (SMD) is a polarizable continuum model that includes the full solute electron density without defining partial atomic charges. It is a universal solvation model, which means that it can be applied to any kind of molecules. [35]. Frequency calculations were performed on the optimized structures to confirm the obtained geometries as true minima by the absence of imaginary frequencies. The optimized structures were further used for molecular electrostatic potential (MESP) and frontier molecular orbital (FMO) analysis on the same level of theory. Chemical reactivity is defined as the tendency of a substance to react chemically with another substance. Reactivity parameters such as electron affinity, ionization energy, chemical softness and hardness, electrophilicity and chemical potential were also calculated. Modeling of the synthesized molecules (**4a**–**4n**) was performed using GaussView 6 [36] and the calculations were performed using the Gaussian 09 [37] program.

#### 2.2.1. Optimized Geometries

**Figure 3:**
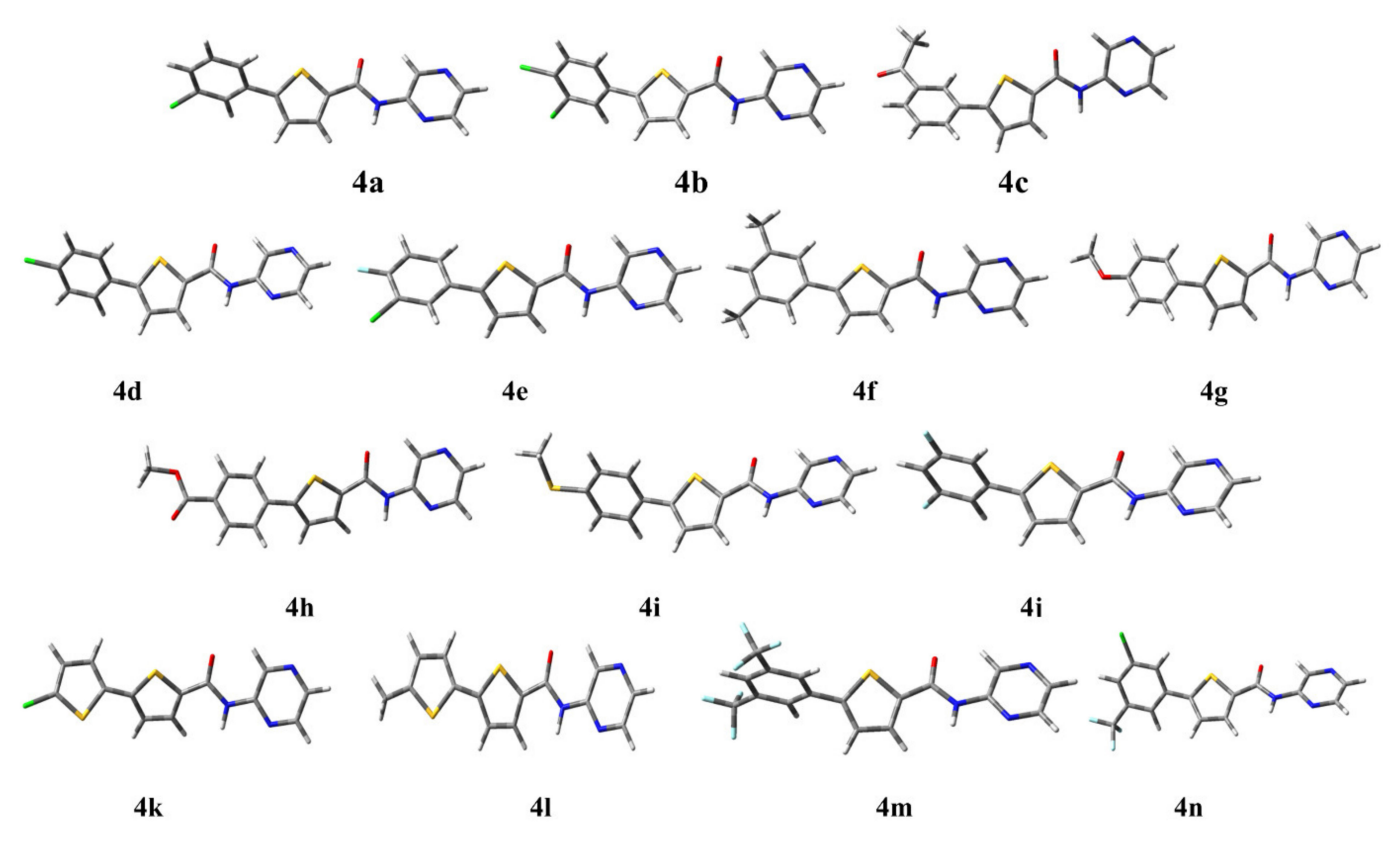
Optimized structures of all the final derivatives (**4a**–**4n**). In the 3D models, the yellow color indicates S, the red color represents oxygen, the grey color indicates C, the green color is for Cl, the light blue color shows F and the white color symbolizes H.

In the field of computational chemistry, geometries optimization is a crucial process to find the ground state geometry of molecules that can be used to compute other properties [38]. In the case of conformationally flexible compounds, a conformational search is crucial to find out the minimum energy conformers that must be used to compute all the other properties. A relaxed potential energy scan was performed considering the important dihedrals, and low-energy conformers were selected from the resultant potential energy scan (PES) followed by geometry optimization and removal of duplicates. These conformers were then subjected to the properties calculations like NMR and Boltzmann averaging of the results was performed afterwards. The Boltzmann-averaged values are provided in the respective tables that show a very good agreement with the experimental data. The optimized geometries of the most stable conformers of the molecules (**4a**–**4n**) calculated at the PBE0-D3BJ/def2-TZVP/SMD_1,4-dioxane_ level of theory are provided in Figure 3. All the PES figures are provided in the Supplementary Materials.

#### 2.2.2. Nuclear Magnetic Resonance Spectroscopy (NMR)

NMR is the most important technique for determining the accurate structure of organic compounds. Quantum calculations are found to be sufficiently accurate in calculating NMR spectra and studying the relationship between the molecular structure and its chemical shifts. Therefore, the use of theoretical methods is very useful for augmenting the confidence in explaining the structure of molecules [39,40,41]. In this study, the experimental ^1^H spectra (**4a**–**4n**) were recorded in 1,4-dioxane while the calculation of the ^1^H and ^13^C NMR spectra was performed using the same level of theory as for the optimizations. The comparison of the experimental and theoretical calculated ^1^H NMR data of compound **4a** is provided in Table 1 while the ^1^H NMR data of the remaining compounds **4b**–**4n** are provided in the Supplementary Materials (Tables S1–S13). It can be seen that the performance of the NMR calculations was very good and the mean absolute error (MAE) is only 0.25 ppm.

#### 2.2.3. FMO and NLO Analysis

The frontier molecular orbitals (FMO) of a compound are the highest occupied molecular orbitals (HOMO) and the lowest unoccupied molecular orbitals (LUMO). Logically, the HOMO are regarded as electron-donating or nucleophilic while the LUMO are regarded as electron-accepting or electrophilic. In addition, chemical reactions and resonances on one or more molecules can be described by overlapping between the filled HOMOs and empty LUMOs. These basic ideas are used in the FMO theory to elucidate the structure and reactivity of compounds. To determine the chemical stability and reactivity of the molecules, the HOMO–LUMO and their energy gap are very important quantum chemical parameters. These MO also have the key role in determining optical and electrical properties.

**Figure 4:**
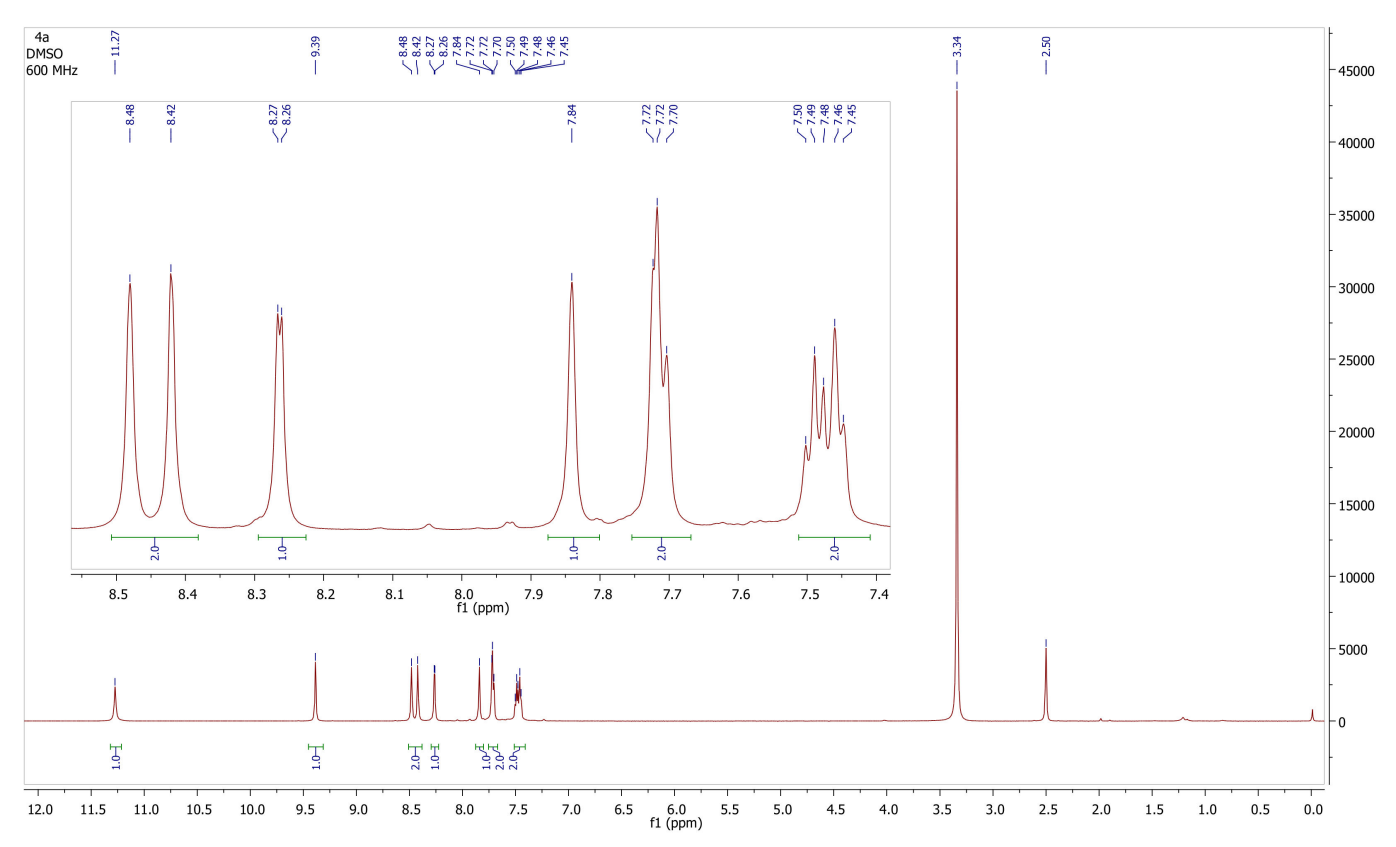
^1^H NMR spectra of compound **4a** at 600 MHz.

The molecular orbitals of all the compounds **4a**–**4n** have almost the same pattern but some compounds have a different one. In most of the compounds, the density of molecular orbitals is mostly spread over the thiophene and benzene rings, but there are some compounds in which the density of molecular orbitals also spreads over the pyrazine ring. A plot of these surfaces is displayed in Figure 4.

The highest HOMO–LUMO energy gap shows that the compound is the most stable and the least reactive and the lowest energy gap shows that the compound is the least stable and the most reactive [42,43,44].

In this series (**4a**–**4n**), it was observed that compound **4m** consisting of three rings (pyrazine, benzene and thiophene) with two CF_3_ substituents and compound **4j** consisting of three rings (pyrazine, benzene and thiophene) with two fluoride substituents have the highest HOMO–LUMO energy gap of 4.93 and 4.89 eV, respectively, which shows that both these compounds are the most stable and the least reactive in this series. Compound **4i** consisting of three rings (pyrazine, benzene and thiophene) with an SH substituent has the lowest HOMO–LUMO energy gap of 4.21 eV which shows that it is the least stable one with the highest reactivity. All the other compounds have a HOMO–LUMO energy gap in the range of 4.21–4.93 eV. The values of HOMO and LUMO energies and their energy gap (ΔE) along with their polarizability (α) and hyperpolarizability (β) of all the compounds **4a**–**4n** are provided in Table 2.

Through experimental and theoretical methods, organic nonlinear optical (NLO) materials have been widely studied to explore their potential applications in optical signal processing, telecommunications and optical data storage. Theoretical research may contribute the key role in understanding the origin of molecular NLO properties and predicting the relationship between the structure and the NLO properties, which is the basis for the design and production of new materials [45]. Most organic compounds with pi-conjugated systems are mainly suitable for the expansion of the NLO material because, among other features, they show large nonlinearity which is due to electronic polarizability.

In this series (**4a**–**4n**), compounds **4i** and **4l** have the highest hyperpolarizability values of 9139.57 a.u. and 9048.28 a.u., respectively, showing the highest NLO responses, while the remaining compounds show very small hyperpolarizability values. Our research group reported N-heterocyclic derivatives having smaller values of hyperpolarizability as compared to the compounds in this study (**4a**–**4n**) [34].

#### 2.2.4. Molecular Electrostatic Potential (MESP) Analysis

In recent years, the maps of molecular electrostatic potential have been extensively used to recognize the reactive sites for nucleophilic and electrophilic attacks during chemical reactions, the study of H-bond interactions and the biological recognition process. MESP is related to the distribution of the total charge of a compound and provides information about some physical properties of the molecules such as partial charges of the atoms, electronegativity, chemical reactivity and dipole moment. In the MESP diagram, different electrostatic potential values on the surface are shown with different colors. The most positive electrostatic potential area is shown in blue, the most negative electrostatic potential area is shown in red, and green is the zero potential area [46,47,48].

**Figure 5:**
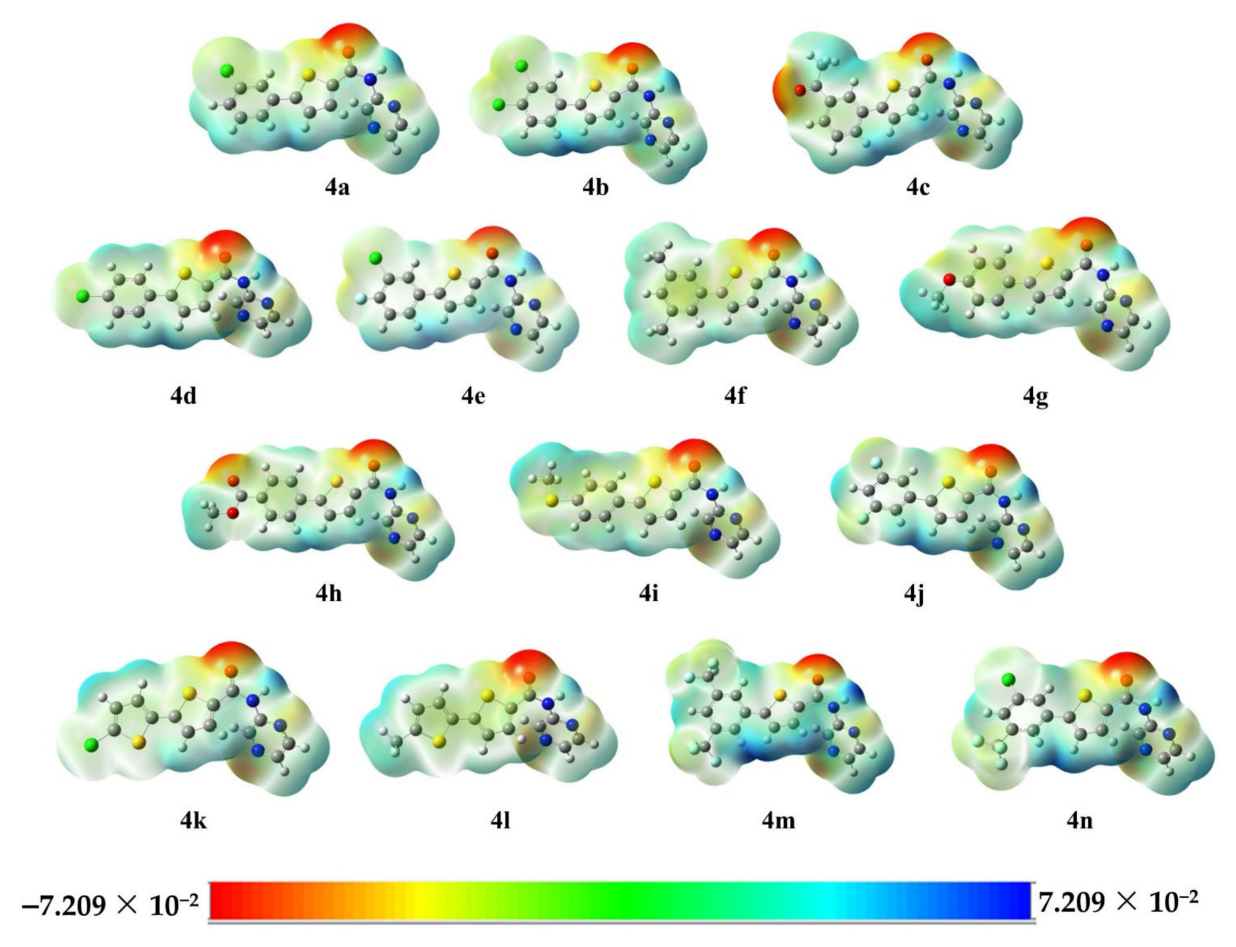
Molecular electrostatic potential surfaces of the final compounds **4a**–**4n** computed at the PBE0-D3BJ/def2-TZVP/SMD_1,4-dioxane_ level of theory. The units for the scale are atomic units.

**Figure 6:**
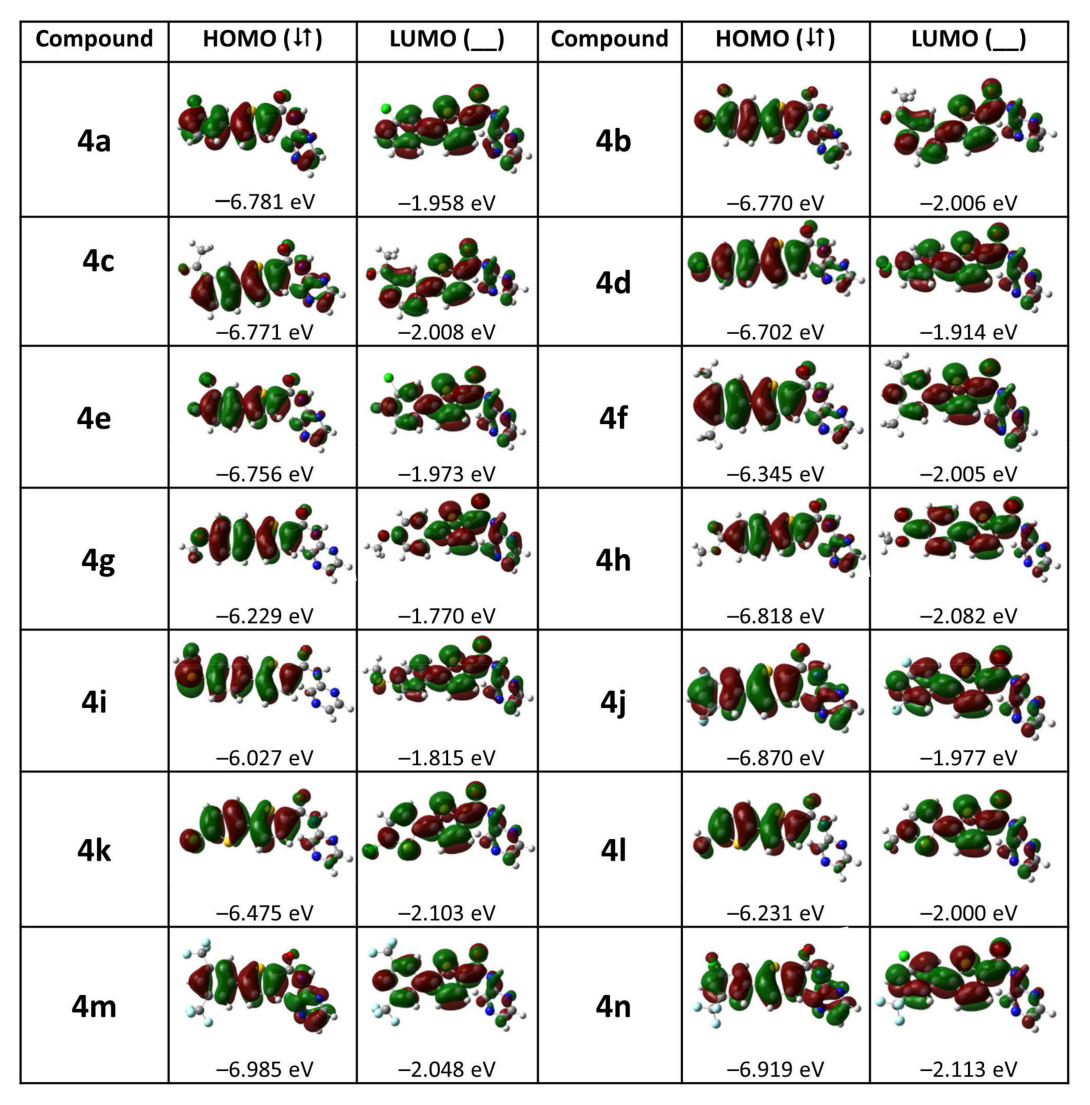
FMO surfaces of compounds **4a**–**4n** computed at the PBE0-D3BJ/def2-TZVP/SMD_1,4-dioxane_ level of theory.

In the maps of the MESP of compounds **4a**–**4n** (Figure 5), the red color shows that the most negative ESP regions are mainly located over the oxygen of the carbonyl group, which is a suitable position for the attack of electrophiles while the blue color shows that the most positive ESP regions are mainly located over the hydrogen of the amide group, which is a suitable position for the attack of nucleophiles. The maps of the MESP of compounds **4a**–**4n** are provided in Figure 6.

#### 2.2.5. Reactivity Descriptors

Chemical reactivity is the key concept because it is closely related to the reaction mechanism so chemical reactions can be understood and synthetic procedures can be improved to obtain new materials. The ionization potential (I = −E_HOMO_) and electron affinity (A = −E_LUMO_) were calculated using Koopman’s theorem [49] as well as directly and a comparison is provided in Table 3.

The electronic chemical potential (μ) and molecular hardness (η) have been proposed to measure global reactivity [50,51]. Chemical potential (μ) describes the charge transfer at the ground state within the compound. The electrophilicity index (ω) is a thermodynamic characteristic that calculates the energy changes of a saturated chemical system after the addition of electrons. It plays an important role in determining the chemical reactivity of a system. The values of η, σ, μ and ω were calculated using the following Equations (1)–(4) [52] and are mentioned in Table 4.

Chemical hardness (η) = (I − A)/2(1)

Chemical softness (σ) = 1/η(2)

Chemical potential (μ) = −(I + A)/2(3)

Electrophilicity index (ω) = μ^2^/2η (4)

The lowest values of ionization energy (5.67 eV) and electron affinity (0.06 eV) of compounds **4i** and **4c** mentioned in Table 4 endorse their high reactivity and lowest stability in series **4a**–**4n**.

Among all the derivatives, **4f** has the lowest value of η (2.46 eV) and the highest value of σ (0.40); thus, it is chemically soft (more reactive), whereas **4c** has the highest value of η (3.56 eV) and the lowest value of σ (0.28 eV) and is a chemically hardest compound (less reactive). These results correlate with the HOMO–LUMO energy gaps of all the synthesized compounds.

Compound **4i** has the highest electronic chemical potential value (−3.02 eV) while **4j** has the lowest chemical potential value (−3.90 eV). The results indicate that **4g** has the lowest electrophilicity index value of 1.69 eV and has nucleophilic nature, whereas **4j** has the highest value, 2.25 eV, and has a strongly electrophilic nature.

## 3. Materials and Methods

### 3.1. General Information

All the chemicals were purchased from Sigma-Aldrich (Burlington, MA, USA), and commercial-grade solvents were used. Melting points of the synthesized compounds were checked with a Buchi B-540 melting point apparatus (New Castle, DE, USA). A Bruker NMR spectrophotometer (Billerica, MA, USA) was used to attain NMR spectra by using DMSO-*d*_6_ and CDCl_3_ solvents. Mass spectra were generated on a JEOL spectrometer JMS-HX-110 (USA). To monitor the reaction’s progress, TLC on silica gel 60 PF_254_ cards (Merck, Kenilworth, NJ, USA) was used. UV light (254–365 nm) was used to visualize the product on TLC. For the purification of compounds, silica gel (70–230 mesh) was used in columns.

### 3.2. Synthetic Procedure for 5-Bromo-N-(pyrazin-2-yl)thiophene-2-carboxamide *(**3**)*

TiCl_4_ (3.0 eq., 43.47 mmol, 4.78 mL) and pyrazin-2-amine (**1**) (1 eq., 14.49 mmol, 1.378 g) were mixed in a solution of 5-bromothiophene-2-carboxylic acid (**2**) (1 eq., 14.49 mmol, 3 g) and pyridine (150 mL). The reaction mixture in a sealed schlenk flask was continuously stirred for 2 h at 85 °C. After cooling, pyridine was separated by coevaporation with toluene. Then, an aqueous solution of 1N HCl (150 mL) was added and removed with DCM (3 × 150 mL). A saturated aqueous solution of NaHCO_3_ (3 × 150 mL) was used to wash the combined organic layers, dried by anhydrous sodium sulfate and evaporated on a rotary evaporator. The final product was obtained after column chromatography (n-hexane/ethyl acetate, 90:10) with 75% yield [53,54].

### 3.3. Synthetic Procedure for 5-Aryl-N-(pyrazin-2-yl)thiophene-2-carboxamide *(**4a***–***4n**)*

In an oven-dried schlenk flask, a 1,4-dioxane (8 mL) solvent, 5-bromo-*N*-(pyrazin-2-yl)thiophene-2-carboxamide (**3**) (1 eq., 0.704 mmol, 0.2 g) with tetrakis(triphenylphosphine)-palladium (7 mol%, 0.057 g) were added at room temperature, and inert atmosphere was applied. After stirring the reaction for half an hour, boronic acid/pinacol esters (1.1 eq., 0.774 mmol), potassium phosphate (2 eq., 1.408 mmol, 0.297 g) and water (2 mL) were added [55,56,57]. The mixture was kept on stirring at reflux for more than 20 h. After cooling to normal temperature, the reaction mixture was filtered with solvent ethyl acetate and then concentrated under reduced pressure. The resulted products were purified with column chromatography (n-hexane/ethyl acetate, 75:25) [58,59,60,61,62,63]. The final products (**4a**–**4n**) were dried, recrystallized and finally analyzed with NMR and mass spectrometry. The NMR spectra of the synthesized compounds are provided in the Supplementary Materials.

### 3.4. Characterization Data

Compound 5-bromo-*N*-(pyrazin-2-yl)thiophene-2-carboxamide (**3**). Off-white solid, MP = 224–226 °C (75% yield, 3.3 g). ^1^H NMR (400 MHz, CDCl_3_): δ 9.62 (s, 1H), 8.40 (s, 1H), 8.29 (d, *J* = 11.3 Hz, 2H), 7.46 (d, *J* = 3.9 Hz, 1H), 7.14 (d, *J* = 3.8 Hz, 1H); ^13^C NMR (100 MHz, CDCl_3_): δ 159.0, 151.1, 150.6, 147.0, 140.5, 130.9, 128.8, 121.4, 119.8, 115.2, 21.5. EI/MS *m/z* (%): 285.1 [M + H]^+^; 286.1 [M + 2]; [M‒Br] = 204.0. Analitically calculated for C_9_H_6_BrN_3_OS: C, 38.04; H, 2.13; N, 14.79. Found: C, 38.11; H, 2.15; N, 14.74.

Compound 5-(3-chlorophenyl)-*N*-(pyrazin-2-yl)thiophene-2-carboxamide (**4a**). Light yellow solid, MP = 187–189 °C (72% yield, 160 mg). ^1^H NMR (600 MHz, DMSO-*d*_6_): δ 11.27 (s, 1H), 9.39 (s, 1H), 8.45 (d, *J* = 35.5 Hz, 2H), 8.26 (d, *J* = 3.4 Hz, 1H), 7.84 (s, 1H), 7.72–7.70 (m, 2H), 7.50–7.45 (m, 2H); ^13^C NMR (151 MHz, DMSO-*d*_6_): δ 160.14, 148.72, 147.66, 142.53, 139.98, 138.25, 137.31, 134.81, 134.04, 131.86, 131.12, 128.55, 125.90, 125.32, 124.60. EI/MS *m/z* (%): 316.8 [M + H]^+^; [M‒Cl] = 280.1. Analitically calculated for C_15_H_10_ClN_3_OS: C, 57.05; H, 3.19; N, 13.31. Found: C, 57.09; H, 3.22; N, 13.26.

Compound 5-(3,4-dichlorophenyl)-*N*-(pyrazin-2-yl)thiophene-2-carboxamide (**4b**). Off-white solid, MP = 191–193 °C (59% yield, 146 mg). ^1^H NMR (600 MHz, DMSO-*d*_6_): δ 11.27 (s, 1H), 9.38 (s, 1H), 8.47 (s, 1H), 8.42 (d, *J* = 2.2 Hz, 1H), 8.25 (d, *J* = 3.9 Hz, 1H), 8.03 (d, *J* = 1.5 Hz, 1H), 7.74–7.69 (m, 3H); ^13^C NMR (151 MHz, DMSO-*d*_6_): δ 160.05, 148.68, 146.47, 142.51, 140.00, 138.63, 137.29, 133.38, 132.07, 131.84, 131.31, 131.18, 127.32, 126.36, 125.99. EI/MS *m/z* (%): 351.2 [M+H]^+^; [M‒2Cl] = 279.3. Analitically calculated for C_15_H_9_Cl_2_N_3_OS: C, 51.44; H, 2.59; N, 12.00. Found: C, 51.47; H, 2.62; N, 11.92.

Compound 5-(3-acetylphenyl)-*N*-(pyrazin-2-yl)thiophene-2-carboxamide (**4c**). Light brown solid, MP = 171–173 °C (60% yield, 137 mg). ^1^H NMR (600 MHz, DMSO-*d*_6_): δ 11.25 (s, 1H), 9.42 (d, *J* = 1.2 Hz, 1H), 8.47 (d, *J* = 31.2 Hz, 2H), 8.17 (dd, *J* = 3.6, 1.2 Hz, 1H), 7.90–7.86 (m, 2H), 7.76 (t, *J* = 8.4 Hz, 1H) 7.62–7.57 (m, 2H), 2.43 (s, 3H); ^13^C NMR (151 MHz, DMSO-*d*_6_): δ 197.95, 160.17, 147.56, 142.59, 139.93, 138.31, 136.96, 134.86, 133.87, 132.41, 130.89, 129.42, 124.20, 27.26. EI/MS *m/z* (%): 324.4 [M + H]^+^; [M‒COCH_3_] = 280.1. Analitically calculated for C_17_H_13_N_3_O_2_S: C, 63.14; H, 4.05; N, 12.99. Found: C, 63.19; H, 4.08; N, 12.97.

Compound 5-(4-chlorophenyl)-*N*-(pyrazin-2-yl)thiophene-2-carboxamide (**4d**). Off-white solid, MP = 249–251 °C (68% yield, 150 mg). ^1^H NMR (600 MHz, DMSO-*d*_6_): δ 11.25 (s, 1H), 9.39 (s, 1H), 8.47 (s, 1H), 8.41 (d, *J* = 2.1 Hz, 1H), 8.25 (d, *J* = 3.8 Hz, 1H), 7.77 (d, *J* = 8.3 Hz, 2H), 7.64 (d, *J* = 3.8 Hz, 1H), 7.51 (d, *J* = 8.3 Hz, 2H); ^13^C NMR (151 MHz, DMSO-*d*_6_): δ 160.16, 148.74, 148.15, 142.50, 139.94, 139.89, 137.84, 137.32, 133.40, 131.94, 130.77, 129.23, 128.38, 127.53, 125.36. EI/MS *m/z* (%): 316.8 [M + H]^+^; [M‒Cl] = 280.1. Analitically calculated for C_15_H_10_ClN_3_OS: C, 57.05; H, 3.19; N, 13.31. Found: C, 57.09; H, 3.23; N, 13.28.

Compound 5-(3-chloro-4-fluorophenyl)-*N*-(pyrazin-2-yl)thiophene-2-carboxamide (**4e**). Light yellow solid, MP = 229–231 °C (50% yield, 116 mg). ^1^H NMR (600 MHz, DMSO-*d*_6_): δ 11.27 (s, 1H), 9.38 (s, 1H), 8.48 (d, *J* = 1.6 Hz, 1H), 8.42 (d, *J* = 2.4 Hz, 1H), 8.25 (d, *J* = 4.0 Hz, 1H), 8.01 (dd, *J* = 6.9, 2.1 Hz, 1H), 7.76 (ddd, *J* = 8.4, 4.4, 2.3 Hz, 1H), 7.68 (d, *J* = 4.0 Hz, 1H), 7.51 (t, *J* = 8.9 Hz, 1H); ^13^C NMR (151 MHz, DMSO-*d*_6_): δ 160.12, 158.13, 156.47, 148.71, 146.81, 142.53, 140.00, 137.30, 131.86, 130.78, 127.75, 126.69, 125.93, 120.62, 117.83. EI/MS *m/z* (%): 334.8 [M + H]^+^; [M‒F, Cl] = 279.3. Analitically calculated for C_15_H_9_ClFN_3_OS: C, 53.98; H, 2.72; N, 12.59. Found: C, 54.05; H, 2.73; N, 12.56.

Compound 5-(3,5-dimethylphenyl)-*N*-(pyrazin-2-yl)thiophene-2-carboxamide (**4f**). Light blue solid, MP = 154–156 °C (62% yield, 135 mg). ^1^H NMR (600 MHz, DMSO-*d*_6_): δ 11.18 (s, 1H), 9.40 (s, 1H), 8.44 (s, 1H), 8.39 (s, 1H), 8.23 (d, *J* = 3.8 Hz, 1H), 7.52 (d, *J* = 3.8 Hz, 1H), 7.32 (s, 2H), 6.97 (s, 1H), 2.28 (s, 6H); ^13^C NMR (151 MHz, DMSO-*d*_6_): δ 160.26, 150.11, 148.83, 142.40, 139.76, 138.33, 137.24, 136.96, 136.05, 135.85, 132.66, 131.89, 130.29, 124.33, 123.55, 20.75. EI/MS *m/z* (%): 310.4 [M + H]^+^; [M‒2CH_3_] = 279.1. Analitically calculated for C_17_H_15_N_3_OS: C, 66.00; H, 4.89; N, 13.58. Found: C, 66.06; H, 4.92; N, 13.55.

Compound 5-(4-methoxyphenyl)-*N*-(pyrazin-2-yl)thiophene-2-carboxamide (**4g**). Light brown solid, MP = 110–112 °C (57% yield, 125 mg). ^1^H NMR (600 MHz, DMSO-*d*_6_): δ 11.19 (s, 1H), 9.33 (d, *J* = 1.8 Hz, 1H), 8.46 (d, *J* = 1.8 Hz, 1H), 8.41 (d, *J* = 2.4 Hz, 1H), 8.24–8.20 (m, 2H), 7.66 (d, *J* = 8.4 Hz, 2H), 7.33 (d, *J* = 8.4 Hz, 2H), 2.50 (s, 3H); ^13^C NMR (151 MHz, DMSO-*d*_6_): δ 160.23, 158.85, 148.89, 147.99, 142.38, 139.81, 138.32, 137.18, 135.49, 132.18, 130.39, 127.47, 113.17, 53.93. EI/MS *m/z* (%): 312.4 [M + H]^+^; [M‒OCH_3_] = 280.1. Analitically calculated for C_16_H_13_N_3_O_2_S: C, 61.72; H, 4.21; N, 13.50. Found: C, 61.78; H, 4.24; N, 13.48.

Compound methyl 4-(5-(pyrazin-2-ylcarbamoyl)thiophen-2-yl)benzoate (**4h**). Brown solid, MP = 144–146 °C (46% yield, 110 mg). ^1^H NMR (600 MHz, DMSO-*d*_6_): δ 11.21 (s, 1H), 9.38 (d, J = 1.2 Hz, 1H), 8.48 (s, 1H), 8.41 (d, *J* = 3.0 Hz, 1H), 8.26 (d, *J* = 4.8 Hz, 1H), 7.94 (d, *J* = 4.8 Hz, 1H), 7.78 (d, *J* = 8.4 Hz, 2H), 7.24 (d, *J* = 8.4 Hz, 2H), 3.89 (s, 3H); ^13^C NMR (151 MHz, DMSO-*d*_6_): δ 161.56, 160.45, 148.79, 142.51, 139.91, 138.55, 137.32, 133.23, 131.47, 130.78, 129.99, 128.40, 127.72, 115.08, 53.81. EI/MS *m/z* (%): 340.4 [M + H]^+^; [M‒COOCH_3_] = 280.1. Analitically calculated for C_17_H_13_N_3_O_3_S: C, 60.17; H, 3.86; N, 12.38. Found: C, 60.24; H, 3.90; N, 12.35.

Compound 5-(4-(methylthio)phenyl)-*N*-(pyrazin-2-yl)thiophene-2-carboxamide (**4i**). Light brown solid, MP = 229–231 °C (52% yield, 120 mg). ^1^H NMR (600 MHz, DMSO-*d*_6_): δ 11.22 (s, 1H), 9.38 (d, *J* = 1.1 Hz, 1H), 8.48–8.47 (m, 1H), 8.41 (d, *J* = 2.4 Hz, 1H), 8.24 (d, *J* = 4.0 Hz, 1H), 7.70 (d, *J* = 8.4 Hz, 2H), 7.60 (d, *J* = 4.0 Hz, 1H), 7.33 (d, *J* = 8.4 Hz, 2H), 2.52 (s, 3H); ^13^C NMR (151 MHz, DMSO-*d*_6_): δ 160.25, 149.40, 148.82, 142.52, 139.87, 139.54, 137.29, 136.78, 132.00, 129.27, 126.28, 126.23, 124.33, 14.43. EI/MS *m/z* (%): 328.4 [M + H]^+^; [M‒SCH_3_] = 280.1. Analitically calculated for C_16_H_13_N_3_OS_2_: C, 58.69; H, 4.00; N, 12.83. Found: C, 58.75; H, 4.02; N, 12.80.

Compound 5-(3,5-difluorophenyl)-*N*-(pyrazin-2-yl)thiophene-2-carboxamide (**4j**). Brown solid, MP = 239–241 °C (58% yield, 130 mg). ^1^H NMR (600 MHz, DMSO-*d*_6_): δ 11.28 (s, 1H), 9.37 (d, *J* = 1.4 Hz, 1H), 8.47 (dd, *J* = 2.4, 1.6 Hz, 1H), 8.26 (d, *J* = 4.0 Hz, 1H), 7.76 (d, *J* = 4.0 Hz, 1H), 7.63–7.60 (m, 1H), 7.52 (dd, *J* = 8.4, 1.9 Hz, 2H), 7.26 (tt, *J* = 9.2 Hz, 2.1 Hz, 1H); ^13^C NMR (151 MHz, DMSO-*d*_6_): δ 163.70, 162.07, 160.06, 148.67, 146.58, 142.53, 140.03, 138.90, 137.29, 136.12, 131.70, 128.67, 126.74, 109.13, 103.93. EI/MS *m/z* (%): 318.4 [M + H]^+^; [M‒2F] = 279.1. Analitically calculated for C_15_H_9_F_2_N_3_OS: C, 56.78; H, 2.86; N, 13.24. Found: C, 56.88; H, 2.89; N, 13.20.

Compound 5′-chloro-*N*-(pyrazin-2-yl)-2,2′-bithiophene-5-carboxamide (**4k**). Off-white solid, MP = 180–182 °C (39% yield, 90 mg). ^1^H NMR (600 MHz, DMSO-*d*_6_): δ 11.26 (s, 1H), 9.42 (d, *J* = 3.0 Hz, 1H), 8.49 (d, *J* = 1.8 Hz, 1H), 8.44 (d, *J* = 2.4 Hz, 1H), 8.28 (d, *J* = 4.2 Hz, 1H), 7.52 (d, *J* = 4.2 Hz, 1H), 7.30 (d, *J* = 6.0 Hz, 1H), 7.12 (d, *J* = 6.0 Hz, 1H), 2.47 (s, 3H); ^13^C NMR (151 MHz, DMSO-*d*_6_): δ 160.22, 148.76, 147.63, 143.45, 141.95, 141.14, 138.07, 136.97, 134.24, 129.84, 128.46, 126.32, 124.84. EI/MS *m/z* (%): 322.8 [M + H]^+^; [M‒Cl] = 286.0. Analitically calculated for C_13_H_8_ClN_3_OS_2_: C, 48.52; H, 2.51; N, 13.06. Found: C, 48.58; H, 2.53; N, 13.00.

Compound 5′-methyl-*N*-(pyrazin-2-yl)-2,2′-bithiophene-5-carboxamide (**4l**). Brown solid, MP = 200–202 °C (43% yield, 96 mg). ^1^H NMR (600 MHz, DMSO-*d*_6_): δ 11.21 (s, 1H), 9.37 (d, *J* = 1.4 Hz, 1H), 8.47 (dd, *J* = 2.4, 1.6 Hz, 1H), 8.41 (d, *J* = 2.5 Hz, 1H), 8.18 (d, *J* = 4.0 Hz, 1H), 7.30 (dd, *J* = 9.1, 3.8 Hz, 2H), 6.86–6.82 (m, 1H), 2.47 (s, 3H); ^13^C NMR (151 MHz, DMSO-*d*_6_): δ 160.11, 148.77, 143.29, 142.51, 141.00, 139.87, 137.25, 135.99, 133.15, 131.86, 127.00, 125.82, 123.98, 14.97. EI/MS *m/z* (%): 302.4 [M + H]^+^; [M‒SCH_3_] = 286.0. Analitically calculated for C_14_H_11_N_3_OS_2_: C, 55.79; H, 3.68; N, 13.94. Found: C, 55.86; H, 3.63; N, 13.96.

Compound 5-(3,5-bis(trifluoromethyl)phenyl)-*N*-(pyrazin-2-yl)thiophene-2-carboxamide (**4m**). Brown solid, MP = 203–205 °C (48% yield, 140 mg). ^1^H NMR (600 MHz, DMSO-*d*_6_): δ 11.28 (s, 1H), 9.09 (s, 1H), 8.61 (s, 1H), 8.49–8.45 (m, 2H), 8.27 8.02 (d, *J* = 1.8 Hz, 2H), 8.13 7.89 (d, *J* = 8.4 Hz, 1H), 8.10 7.71 (d, *J* = 8.4 Hz, 1H); ^13^C NMR (151 MHz, DMSO-*d*_6_): δ 160.18, 148.73, 147.05, 142.49, 139.93, 138.29, 134.47, 134.01, 133.86, 132.41, 130.19, 129.03, 125.93, 119.97. EI/MS *m/z* (%): 418.4 [M + H]^+^; [M‒6F] = 303.1. Analitically calculated for C_17_H_9_F_6_N_3_OS: C, 48.93; H, 2.17; N, 10.07. Found: C, 48.99; H, 2.19; N, 10.11.

Compound 5-(3-chloro-5-(trifluoromethyl)phenyl)-*N*-(pyrazin-2-yl)thiophene-2-carboxamide (**4n**). Brown solid, MP = 170–172 °C (37% yield, 100 mg). ^1^H NMR (600 MHz, DMSO-*d*_6_): δ 11.24 (s, 1H), 9.13 (s, 1H), 8.54–8.48 (m, 3H), 8.04–8.00 (m, 2H), 7.85 (d, *J* = 7.8 Hz, 1H), 8.01 7.66 (d, *J* = 7.8 Hz, 1H); ^13^C NMR (151 MHz, DMSO-*d*_6_): δ 160.16, 148.77, 147.76, 142.51, 139.89, 138.31, 137.29, 135.41, 134.89, 134.06, 132.21, 130.43, 129.23, 124.87, 119.93. EI/MS *m/z* (%): 384.8 [M + H]^+^; [M‒3F] = 326.0, [M‒Cl] = 348.0. Analitically calculated for C_16_H_9_ClF_3_N_3_OS: C, 50.07; H, 2.36; N, 10.95. Found: C, 50.15; H, 2.38; N, 10.89.

## 4. Conclusions

In this study, 5-bromo-*N*-(pyrazin-2-yl)thiophene-2-carboxamide (**3**) was synthesized by means of TiCl_4_-mediated one-pot condensation of pyrazin-2-amine (**1**) and 5-bromothiophene-2-carboxylic acid (**2**) with a good 75% yield followed by a Suzuki cross-coupling reaction with various aryl/heteroaryl boronic acids/pinacol esters to synthesize 5-aryl-*N*-(pyrazin-2-yl)thiophene-2-carboxamides (**4a**–**4n**) obtained with moderate and good yields (37–72%). The target pyrazine analogs (**4a**–**4n**) were confirmed by NMR and mass spectrometry. The computational studies of these final compounds were also performed to get optimized geometries and thermodynamic parameters such as FMOs (E_HOMO_, E_LUMO_), HOMO–LUMO energy gap, electron affinity (A), ionization energy (I), electrophilicity index (ω), chemical softness (σ) and chemical hardness (η). The stability and NLO behavior of all the compounds **4a**–**4n** were studied with the help of the HOMO–LUMO energy gap and hyperpolarizability calculations. Compound **4i** was found to be the most reactive compound with the highest electronic chemical potential value (−3.88 eV) and compound **4l** had the highest hyperpolarizability value of 8583.80 Hartrees. The theoretical calculated chemical shifts of ^1^H NMR of all compounds **4a**–**4n** match very well the experimental values.

## Figures and Tables

**Table 1:** Comparison of the experimental and theoretical ^1^H NMR data of compound **4a**. The data for the rest of the compounds is provided in the Supplementary Materials. The mean absolute deviation is presented as the mean absolute error and, similarly, the root-mean-square deviation is denoted as the root-mean-square error.

Compound 4a				
Carbon No.	Carbon Type	^1^H NMR (δ, ppm), Experimental	^1^H NMR (δ, ppm), Computed	Δδ, ppm
2	C	–	–	–
3	CH	9.39	10.55	−1.16
4	N	–	–	–
5	CH	8.45	8.43	0.02
6	CH	8.45	8.42	0.03
2′	C	–	–	–
3′	CH	7.84	7.24	0.60
4′	CH	7.72	6.80	0.92
5′	C	–	–	–
1″	C	–	–	–
2″	CH	8.26	7.45	0.81
3″	C	–	–	–
4″	CH	7.50	7.37	0.13
5″	CH	7.45	7.39	0.06
6″	CH	7.70	7.25	0.45

Mean absolute error (MAE) = 0.17; root-mean-square error (RMSE) = 0.37.

**Table 2:** Energies of the HOMO, LUMO, HOMO–LUMO energy gap, polarizability (α) and hyperpolarizability (βo) values of compounds **4a**–**4n**.

Compounds	E_HOMO_ (eV)	E_LUMO_ (eV)	E_LUMO_−E_HOMO_ (eV)	α (a.u.)	βo (a.u.)
**4a**	−6.781	−1.958	4.823	310.96	2700.54
**4b**	−6.770	−2.006	4.763	325.22	4139.08
**4c**	−6.771	−2.008	4.763	338.09	2571.25
**4d**	−6.702	−1.914	4.788	311.83	3958.52
**4e**	−6.756	−1.973	4.784	310.60	4004.42
**4f**	−6.345	−2.005	4.340	354.10	6120.41
**4g**	−6.229	−1.770	4.459	330.84	8398.11
**4h**	−6.818	−2.082	4.736	345.57	1363.79
**4i**	−6.027	−1.815	4.212	349.43	9139.57
**4j**	−6.870	−1.977	4.893	293.78	1926.82
**4k**	−6.475	−2.103	4.373	310.21	6520.24
**4l**	−6.231	−2.000	4.231	325.48	9048.28
**4m**	−6.985	−2.048	4.937	315.57	1103.20
**4n**	−6.919	−2.113	4.806	322.84	1416.47

**Table 3:** Ionization potential (I) and electron affinity (A) of compounds **4a**–**4n** calculated through Koopman’s theorem and directly, i.e., through optimization of charged molecules.

Compounds	Calculated through Koopman’s Theorem		Calculated Directly from Charged Molecules	
	I (eV)	A (eV)	I (eV)	A (eV)
**4a**	6.78	1.96	6.16	0.47
**4b**	6.77	2.01	6.23	0.50
**4c**	6.77	2.01	7.19	0.06
**4d**	6.70	1.91	6.16	0.44
**4e**	6.76	1.97	6.23	0.50
**4f**	6.35	2.00	5.75	0.83
**4g**	6.23	1.77	5.88	0.28
**4h**	6.82	2.08	7.20	0.42
**4i**	6.03	1.81	5.67	0.37
**4j**	6.87	1.98	7.29	0.52
**4k**	6.48	2.10	6.21	0.75
**4l**	6.23	2.00	5.79	0.73
**4m**	6.99	2.05	6.61	0.58
**4n**	6.92	2.11	6.36	0.60

**Table 4:** Reactivity descriptors η, σ, μ and ω values of the final compounds **4a**–**4n**.

Compounds	η (eV)	σ (eV^−1^)	μ (eV)	*ω* (eV)
**4a**	2.84	0.35	−3.31	1.93
**4b**	2.86	0.34	−3.36	1.97
**4c**	3.56	0.28	−3.62	1.84
**4d**	2.86	0.34	−3.30	1.90
**4e**	2.86	0.34	−3.36	1.97
**4f**	2.46	0.40	−3.29	2.20
**4g**	2.80	0.35	−3.08	1.69
**4h**	3.39	0.29	−3.81	2.14
**4i**	2.65	0.37	−3.02	1.72
**4j**	3.38	0.29	−3.90	2.25
**4k**	2.73	0.36	−3.48	2.21
**4l**	2.53	0.39	−3.26	2.10
**4m**	3.01	0.33	−3.59	2.14
**4n**	2.88	0.34	−3.48	2.10

## Data Availability

Data are contained within the article and the Supplementary Materials.

## Supplementary Materials

The following are available online. Theoratical ^1^HNMR (Tables S1–S13), the ^1^HNMR and ^13^CNMR (Figures S1–S17), Potential energy Scan (Figures S18–S30) and lowest energy conformers are available online.
