# Supplementary material for: Facile Synthesis of 5-Aryl-N-(pyrazin-2-yl)thiophene-2-carboxamides via Suzuki Cross-Coupling Reactions, Their Electronic and Nonlinear Optical Properties through DFT Calculations
Source: Molecules. 2021 Dec 2;26(23):7309. doi: 10.3390/molecules26237309 (PMC8659105; doi:10.3390/molecules26237309)
Supplement: Supplementary file 1 [file molecules-26-07309-s001.zip › molecules-1362947-supplementary.pdf]

Supplementary Data

Facile Synthesis of 5-aryl-*N*-(pyrazin-2-yl)thiophene-2-carbox-amides via Suzuki Cross-Coupling Reactions, Their Electronic and Nonlinear Optical Properties through DFT Calculations

Gulraiz Ahmad [1], Nasir Rasool [1,*], Adeel Mubarik [1], Ameer Fawad Zahoor [1], Muhammad Ali Hashmi [2], Muhammad Zubair [1], Muhammad Bilal [1], Mohamed Hussien [3], Muhammad Saeed Akhtar [4] and Sajjad Haider [5,*]

[1] Department of Chemistry, Government College, University Faisalabad, Faisalabad 38000, Pakistan;

[2] Department of Chemistry, University of Education, Attock Campus, Attock 43600, Pakistan;

[3] Department of Chemistry, Faculty of Science, King Khalid University, P.O. Box 9004, Abha 61413, Saudi Arabia;

[4] School of Chemical Engineering, Yeungnam University, Gyeongsan 38541, Korea;

[5] Chemical Engineering Department, College of Engineering, King Saud University, P.O.Box 800, Riyadh 11421, Saudi Arabia.

* Corresponding authors E-mail addresses:

Nasir Rasool Email: nasirrasool@gcuf.edu.pk

Sajjad Haider Email: shaider@ksu.edu.sa

Table of Contents

Table S1: Comparison of experimental and theoretical ^{1}H-NMR data of compound 4b S4
Table S2: Comparison of experimental and theoretical ^{1}H-NMR data of compound 4c........................... S5
Table S3: Comparison of experimental and theoretical ^{1}H-NMR data of compound 4d S6
Table S4: Comparison of experimental and theoretical ^{1}H-NMR data of compound 4e........................... S7
Table S5: Comparison of experimental and theoretical ^{1}H-NMR data of compound 4f S8
Table S6: Comparison of experimental and theoretical ^{1}H-NMR data of compound 4g........................... S9
Table S7: Comparison of experimental and theoretical ^{1}H-NMR data of compound 4h S10
Table S8: Comparison of experimental and theoretical ^{1}H-NMR data of compound 4i.......................... S11
Table S9: Comparison of experimental and theoretical ^{1}H-NMR data of compound 4j.......................... S12
Table S10: Comparison of experimental and theoretical ^{1}H-NMR data of compound 4k....................... S13
Table S11: Comparison of experimental and theoretical ^{1}H-NMR data of compound 4l........................ S14
Table S12: Comparison of experimental and theoretical ^{1}H-NMR data of compound 4m S15
Table S13: Comparison of experimental and theoretical ^{1}H-NMR data of compound 4n S16
Figure S1: ^{1}H NMR (400 MHz, $CDCl_3$) of compound 3. .... S17
Figure S2: ^{1}H NMR (600 MHz, DMSO-d_6) of compound 4a. S18
Figure S3: ^{13}C NMR (151 MHz, DMSO-d_6) of compound 4a. S19
Figure S4: ^{1}H NMR (600 MHz, DMSO-d_6) of compound 4b. S20
Figure S5: ^{13}C NMR (151 MHz, DMSO-d_6) of compound 4b. S21
Figure S6: ^{1}H NMR (600 MHz, DMSO-d_6) of compound 4d. S22
Figure S7: ^{13}C NMR (151 MHz, DMSO-d_6) of compound 4d. S23
Figure S8: ^{1}H NMR (600 MHz, DMSO-d_6) of compound 4e. S24
Figure S9: ^{13}C NMR (151 MHz, DMSO-d_6) of compound 4e. S25
Figure S10: ^{1}H NMR (600 MHz, DMSO-d_6) of compound 4f. S26
Figure S11: ^{13}C NMR (151 MHz, DMSO-d_6) of compound 4f. S27
Figure S12: ^{1}H NMR (600 MHz, DMSO-d_6) of compound 4i. S28
Figure S13: ^{13}C NMR (151 MHz, DMSO-d_6) of compound 4i. S29
Figure S14: ^{1}H NMR (600 MHz, DMSO-d_6) of compound 4j. S30
Figure S15: ^{13}C NMR (151 MHz, DMSO-d_6) of compound 4j. S31
Figure S16: ^{1}H NMR (600 MHz, DMSO-d_6) of compound 4l. S32
Figure S17: ^{13}C NMR (151 MHz, DMSO-d_6) of compound 4l. S33
Figure S18. Potential Energy Scan of compound 4a S34

Figure S19. Potential Energy Scan of compound 4b S35
Figure S20. Potential Energy Scan of compound 4c S36
Figure S21. Potential Energy Scan of compound 4d S37
Figure S22. Potential Energy Scan of compound 4e........ S38
Figure S23. Potential Energy Scan of compound 4g........ S39
Figure S24. Potential Energy Scan of compound 4h S40
Figure S25. Potential Energy Scan of compound 4i S41
Figure S26. Potential Energy Scan of compound 4j S42
Figure S27. Potential Energy Scan of compound 4k........ S43
Figure S28. Potential Energy Scan of compound 4l S44
Figure S29. Potential Energy Scan of compound 4m S45
Figure S30. Potential Energy Scan of compound 4n S46
XYZ Coordinates of the lowest energy conformer of 4a S47
XYZ Coordinates of the lowest energy conformer of 4b S48
XYZ Coordinates of the lowest energy conformer of 4c S49
XYZ Coordinates of the lowest energy conformer of 4d S50
XYZ Coordinates of the lowest energy conformer of 4e S51
XYZ Coordinates of the lowest energy conformer of 4f S52
XYZ Coordinates of the lowest energy conformer of 4g S53
XYZ Coordinates of the lowest energy conformer of 4h S54
XYZ Coordinates of the lowest energy conformer of 4i S55
XYZ Coordinates of the lowest energy conformer of 4j S56
XYZ Coordinates of the lowest energy conformer of 4k S57
XYZ Coordinates of the lowest energy conformer of 4l S58
XYZ Coordinates of the lowest energy conformer of 4m S59
XYZ Coordinates of the lowest energy conformer of 4n S60

Table S1: Comparison of experimental and theoretical ^{1}H-NMR data of compound 4b

Compound **4b**				
Carbon No.	Carbon Type	^{1}H-NMR (δ, ppm) Experimental	^{1}H-NMR (δ, ppm) Computed	Δδ, ppm
2	C	-	-	-
3	CH	9.38	10.59	-1.21
4	N	-	-	-
5	CH	8.47	8.44	0.03
6	CH	8.42	8.42	0.00
2'	C	-	-	-
3'	CH	8.25	7.22	1.03
4'	CH	8.03	6.77	1.26
5'	C	-	-	-
1"	C	-	-	-
2"	CH	7.74	7.58	0.16
3"	C	-	-	
4"	C	-	-	-
5"	CH	7.69	7.55	0.14
6"	CH	7.72	7.24	0.48
Mean Absolute Error (MAE) = 0.18				
Root Mean Square Error (RMSE) = 0.42				

Table S2: Comparison of experimental and theoretical ^{1}H-NMR data of compound 4c

Compound **4c**				
Carbon No.	Carbon Type	^{1}H-NMR (δ, ppm) Experimental	^{1}H-NMR (δ, ppm) Computed	Δδ, ppm
2	C	-	-	-
3	CH	9.42	10.64	-1.22
4	N	-	-	-
5	CH	8.17	8.44	-0.27
6	CH	8.45	8.42	0.03
2'	C	-	-	-
3'	CH	7.90	7.26	0.64
4'	CH	7.86	6.82	1.04
5'	C	-	-	-
1"	C	-	-	-
2"	CH	8.49	7.85	0.64
3"	C	-	-	-
4"	CH	7.76	7.89	-0.13
5"	CH	7.56	7.54	0.02
6"	CH	7.62	7.62	0.00
Mean Absolute Error (MAE) = 0.17				
Root Mean Square Error (RMSE) = 0.38				

Table S3: Comparison of experimental and theoretical ^{1}H-NMR data of compound 4d

Compound **4d**				
Carbon No.	Carbon Type	^{1}H-NMR (δ, ppm) Experimental	^{1}H-NMR (δ, ppm) Computed	Δδ, ppm
2	C	-	-	-
3	CH	9.39	10.57	-1.18
4	N	-	-	-
5	CH	8.41	8.43	-0.02
6	CH	8.47	8.42	0.05
2'	C	-	-	-
3'	CH	8.25	7.22	1.03
4'	CH	7.64	6.72	0.92
5'	C	-	-	-
1"	C	-	-	-
2"	CH	7.51	7.39	0.12
3"	CH	7.77	7.41	0.36
4"	C	-	-	-
5"	CH	7.77	7.41	0.36
6"	CH	7.51	7.39	0.12
Mean Absolute Error (MAE) = 0.17				
Root Mean Square Error (RMSE) = 0.38				

Table S4: Comparison of experimental and theoretical ^{1}H-NMR data of compound 4e

Compound **4e**				
Carbon No.	Carbon Type	^{1}H-NMR (δ, ppm) Experimental	^{1}H-NMR (δ, ppm) Computed	Δδ, ppm
2	C	-	-	-
3	CH	9.38	10.63	-1.25
4	N	-	-	-
5	CH	8.42	8.44	-0.02
6	CH	8.48	8.42	0.06
2'	C	-	-	-
3'	CH	8.25	7.22	1.04
4'	CH	8.01	6.77	1.25
5'	C	-	-	-
1"	C	-	-	-
2"	CH	7.76	7.57	0.19
3"	C	-	-	-
4"	C	-	-	-
5"	CH	7.51	7.27	0.24
6"	CH	7.68	7.32	0.36

Mean Absolute Error (MAE) = 0.18

Root Mean Square Error (RMSE) = 0.42

Table S5: Comparison of experimental and theoretical ^{1}H-NMR data of compound 4f

Compound **4f**				
Carbon No.	Carbon Type	^{1}H-NMR (δ, ppm) Experimental	^{1}H-NMR (δ, ppm) Computed	Δδ, ppm
2	C	-	-	-
3	CH	9.40	10.63	-1.23
4	N	-	-	-
5	CH	8.39	8.40	-0.01
6	CH	8.44	8.40	0.04
2'	C	-	-	-
3'	CH	8.23	7.20	1.03
4'	CH	7.52	7.14	0.38
5'	C	-	-	-
1"	C	-	-	-
2"	CH	7.32	7.37	-0.05
3"	C	-	-	-
4"	CH	6.97	7.15	-0.18
5"	C	-	-	-
6"	CH	7.32	7.35	-0.03
Mean Absolute Error (MAE) = 0.12				
Root Mean Square Error (RMSE) = 0.33				

Table S6: Comparison of experimental and theoretical ^{1}H-NMR data of compound 4g

Compound **4g**				
Carbon No.	Carbon Type	^{1}H-NMR (δ, ppm) Experimental	^{1}H-NMR (δ, ppm) Computed	Δδ, ppm
2	C	-	-	-
3	CH	9.33	10.58	-1.25
4	N	-	-	-
5	CH	8.41	8.39	0.02
6	CH	8.46	8.40	0.06
2'	C	-	-	-
3'	CH	8.26	7.18	1.08
4'	CH	8.22	6.65	1.57
5'	C	-	-	-
1"	C	-	-	-
2"	CH	7.66	7.42	0.24
3"	CH	7.33	6.58	0.75
4"	C	-	-	-
5"	CH	7.33	7.19	0.14
6"	CH	7.66	7.43	0.23
Mean Absolute Error (MAE) = 0.22				
Root Mean Square Error (RMSE) = 0.49				

Table S7: Comparison of experimental and theoretical ^{1}H-NMR data of compound 4h

Compound **4h**				
Carbon No.	Carbon Type	^{1}H-NMR (δ, ppm) Experimental	^{1}H-NMR (δ, ppm) Computed	Δδ, ppm
2	C	-	-	-
3	CH	9.38	10.58	-1.20
4	N	-	-	-
5	CH	8.41	8.44	-0.03
6	CH	8.48	8.42	0.06
2'	C	-	-	-
3'	CH	8.26	7.25	1.01
4'	CH	7.94	6.82	1.12
5'	C	-	-	-
1"	C	-	-	-
2"	CH	7.24	7.57	-0.33
3"	CH	7.78	8.33	-0.55
4"	C	-	-	-
5"	CH	7.78	8.54	-0.76
6"	CH	7.24	7.52	-0.28
Mean Absolute Error (MAE) = 0.22				
Root Mean Square Error (RMSE) = 0.44				

Table S8: Comparison of experimental and theoretical ^{1}H-NMR data of compound 4i

Compound **4i**				
Carbon No.	Carbon Type	^{1}H-NMR (δ, ppm) Experimental	^{1}H-NMR (δ, ppm) Computed	Δδ, ppm
2	C	-	-	-
3	CH	9.38	10.57	-1.19
4	N	-	-	-
5	CH	8.41	8.40	0.01
6	CH	8.47	8.40	0.07
2'	C	-	-	-
3'	CH	8.24	7.19	1.05
4'	CH	7.70	6.66	1.04
5'	C	-	-	-
1"	C	-	-	-
2"	CH	7.70	7.33	0.37
3"	CH	7.33	6.87	0.46
4"	C	-	-	-
5"	CH	7.33	7.20	0.13
6"	CH	7.60	7.30	0.30
Mean Absolute Error (MAE) = 0.19<br>Root Mean Square Error (RMSE) = 0.41				

Table S9: Comparison of experimental and theoretical ^{1}H-NMR data of compound 4j

Compound **4j**				
Carbon No.	Carbon Type	^{1}H-NMR (δ, ppm) Experimental	^{1}H-NMR (δ, ppm) Computed	Δδ, ppm
2	C	-	-	-
3	CH	9.37	10.52	-1.15
4	N	-	-	-
5	CH	8.26	8.44	-0.18
6	CH	8.47	8.43	0.04
2'	C	-	-	-
3'	CH	7.76	7.25	0.51
4'	CH	7.61	6.82	0.79
5'	C	-	-	-
1"	C	-	-	-
2"	CH	7.52	6.92	0.60
3"	C	-	-	-
4"	CH	7.26	6.95	0.31
5"	C	-	-	-
6"	CH	7.52	6.95	0.57
Mean Absolute Error (MAE) = 0.17				
Root Mean Square Error (RMSE) = 0.35				

Table S10: Comparison of experimental and theoretical ^{1}H-NMR data of compound 4k

Compound **4k**				
Carbon No.	Carbon Type	^{1}H-NMR (δ, ppm) Experimental	^{1}H-NMR (δ, ppm) Computed	Δδ, ppm
2	C	-	-	-
3	CH	9.42	10.56	-1.14
4	N	-	-	-
5	CH	8.44	8.43	0.01
6	CH	8.49	8.41	0.08
2'	C	-	-	-
3'	CH	8.28	7.19	1.09
4'	CH	7.52	6.73	0.79
5'	C	-	-	-
1''	C	-	-	-
2''	C	-	-	-
3''	CH	7.30	6.78	0.52
4''	CH	7.12	6.63	0.49
5''	C	-	-	-
Mean Absolute Error (MAE) = 0.17<br>Root Mean Square Error (RMSE) = 0.38				

Table S11: Comparison of experimental and theoretical ^{1}H-NMR data of compound 4l

Compound **4l**				
Carbon No.	Carbon Type	^{1}H-NMR (δ, ppm) Experimental	^{1}H-NMR (δ, ppm) Computed	Δδ, ppm
2	C	-	-	-
3	CH	9.37	10.57	-1.20
4	N	-	-	-
5	CH	8.41	8.40	0.01
6	CH	8.47	8.40	0.07
2'	C	-	-	-
3'	CH	8.18	7.15	1.04
4'	CH	7.30	6.71	0.59
5'	C	-	-	-
1''	C	-	-	-
2''	C	-	-	-
3''	CH	6.84	6.76	0.08
4''	CH	7.30	6.46	0.84
5''	C	-	-	-
Mean Absolute Error (MAE) = 0.16				
Root Mean Square Error (RMSE) = 0.38				

Table S12: Comparison of experimental and theoretical ^{1}H-NMR data of compound 4m

Compound **4m**				
Carbon No.	Carbon Type	^{1}H-NMR (δ, ppm) Experimental	^{1}H-NMR (δ, ppm) Computed	Δδ, ppm
2	C	-	-	-
3	CH	9.09	10.56	-1.47
4	N	-	-	-
5	CH	8.49	8.48	0.01
6	CH	8.61	8.45	0.16
2'	C	-	-	-
3'	CH	7.89	7.28	0.61
4'	CH	7.71	6.85	0.86
5'	C	-	-	-
1"	C	-	-	-
2"	CH	8.02	8.01	0.01
3"	C	-	-	-
4"	CH	8.45	8.23	0.22
5"	C	-	-	-
6"	CH	8.02	8.06	-0.04
Mean Absolute Error (MAE) = 0.14				
Root Mean Square Error (RMSE) = 0.37				

Table S13: Comparison of experimental and theoretical ^{1}H-NMR data of compound 4n

Compound **4n**				
Carbon No.	Carbon Type	^{1}H-NMR (δ, ppm) Experimental	^{1}H-NMR (δ, ppm) Computed	Δδ, ppm
2	C	-	-	-
3	CH	9.13	10.57	-1.44
4	N	-	-	-
5	CH	8.51	8.46	0.05
6	CH	8.54	8.44	0.10
2'	C	-	-	-
3'	CH	8.00	7.28	0.72
4'	CH	7.85	6.88	0.97
5'	C	-	-	-
1"	C	-	-	-
2"	CH	8.48	7.68	0.80
3"	C	-	-	-
4"	CH	7.66	7.74	-0.08
5"	C	-	-	-
6"	CH	8.04	7.69	0.36
Mean Absolute Error (MAE) = 0.19<br>Root Mean Square Error (RMSE) = 0.42				

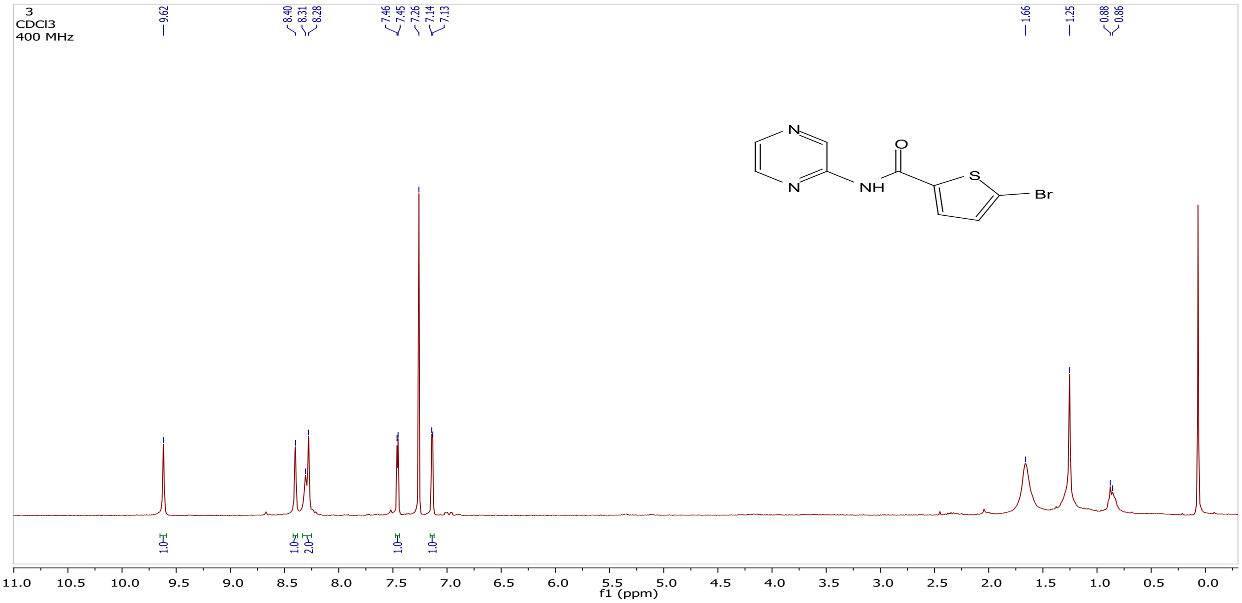

Figure S1: ^{1}H NMR (400 MHz, $CDCl_3$) of compound 3.

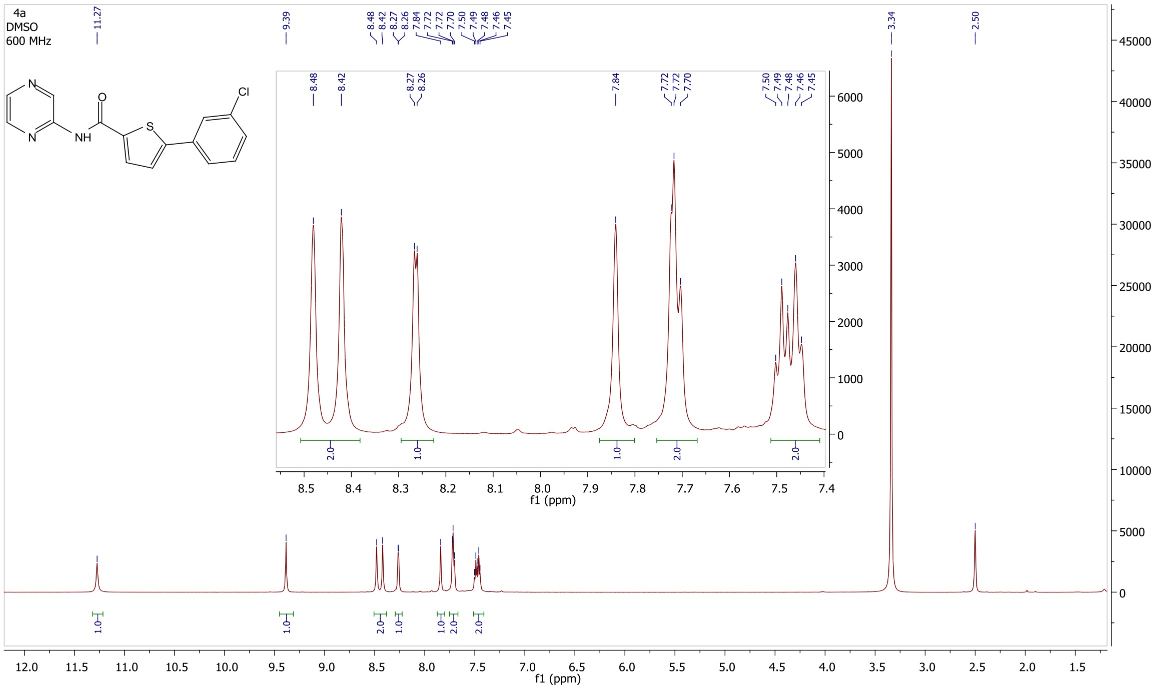

Figure S2: ^{1}H NMR (600 MHz, DMSO-d$_6$) of compound 4a.

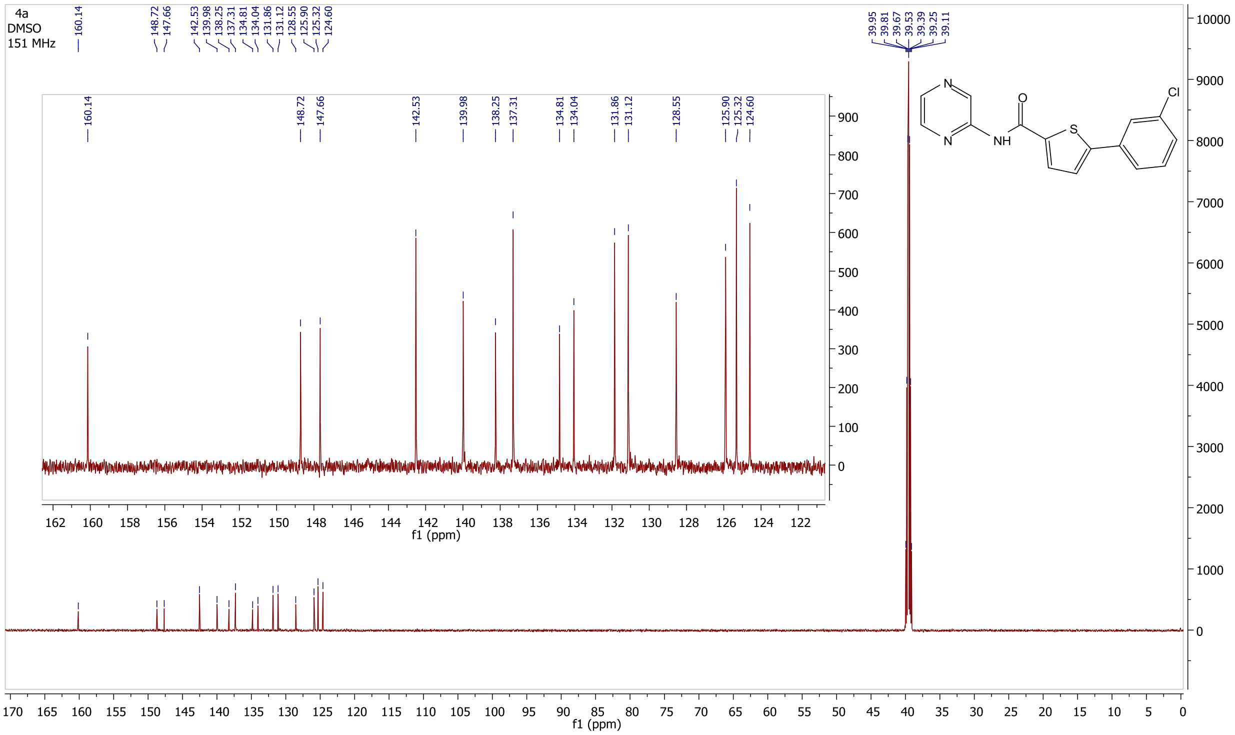

Figure S3: ^{13}C NMR (151 MHz, DMSO-d$_6$) of compound 4a.

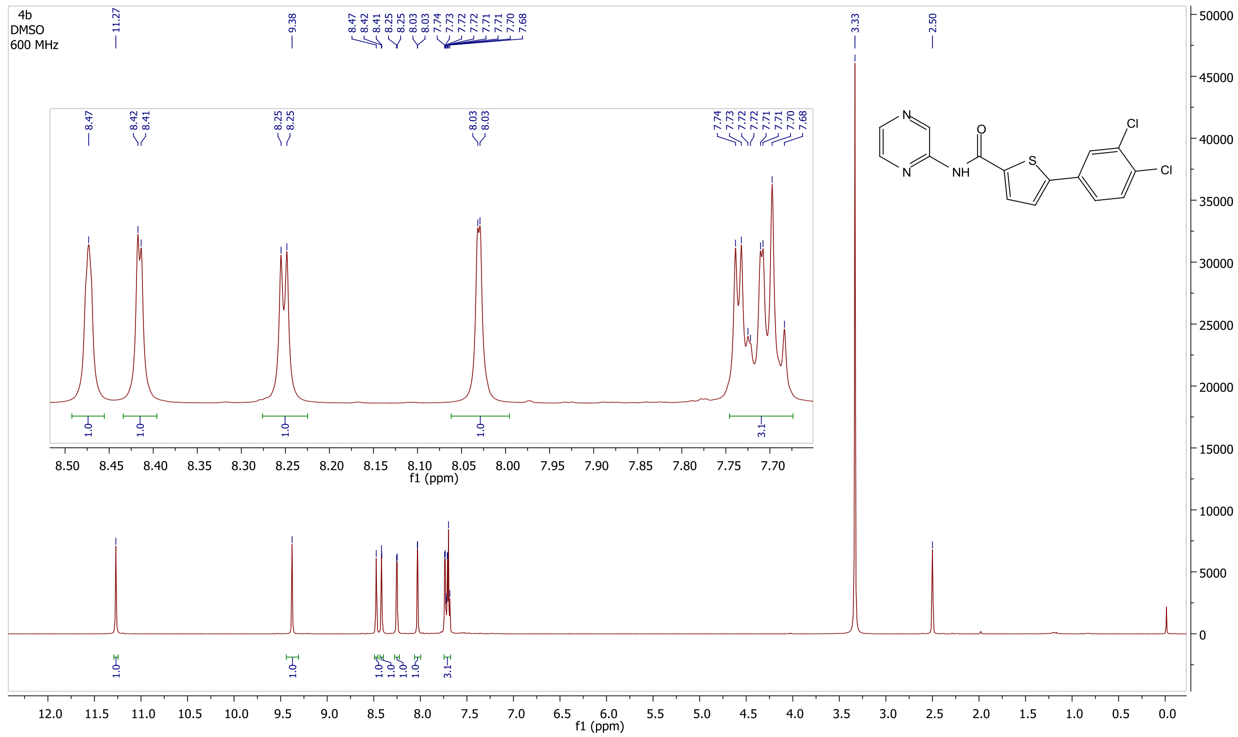

Figure S4: ^{1}H NMR (600 MHz, DMSO-d$_6$) of compound 4b.

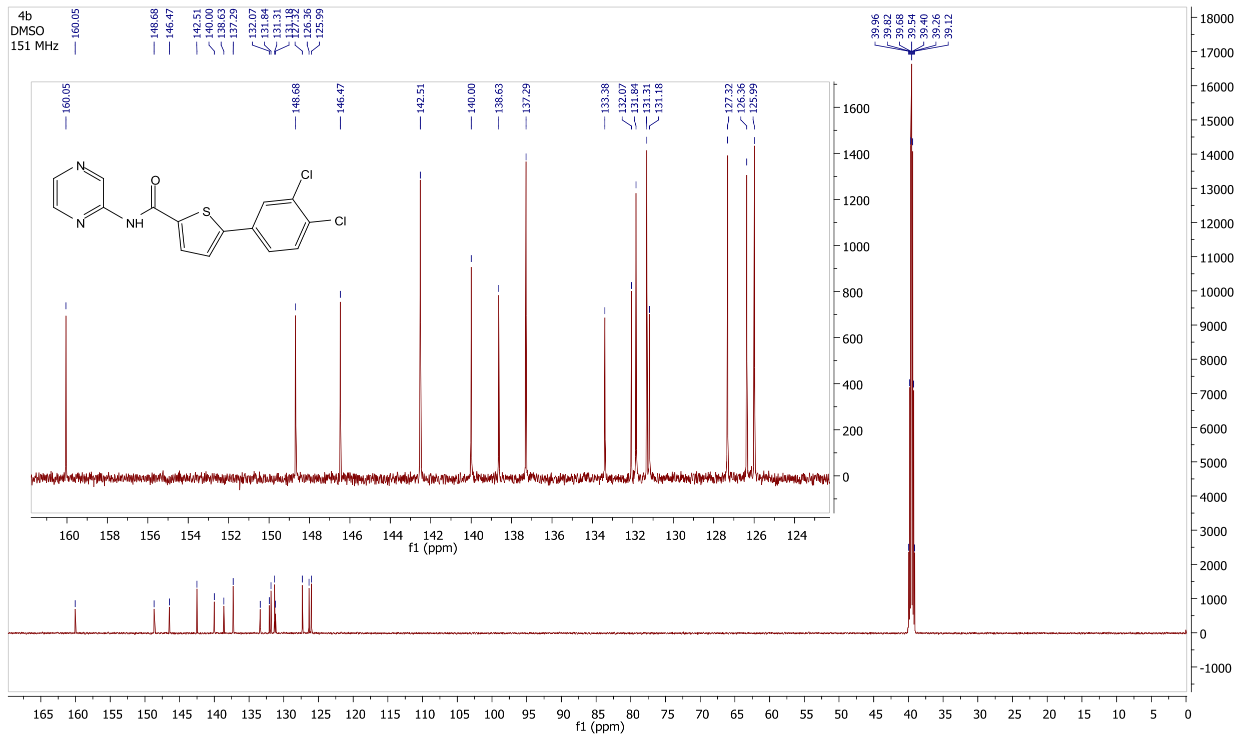

Figure S5: ^{13}C NMR (151 MHz, DMSO-d$_6$) of compound 4b.

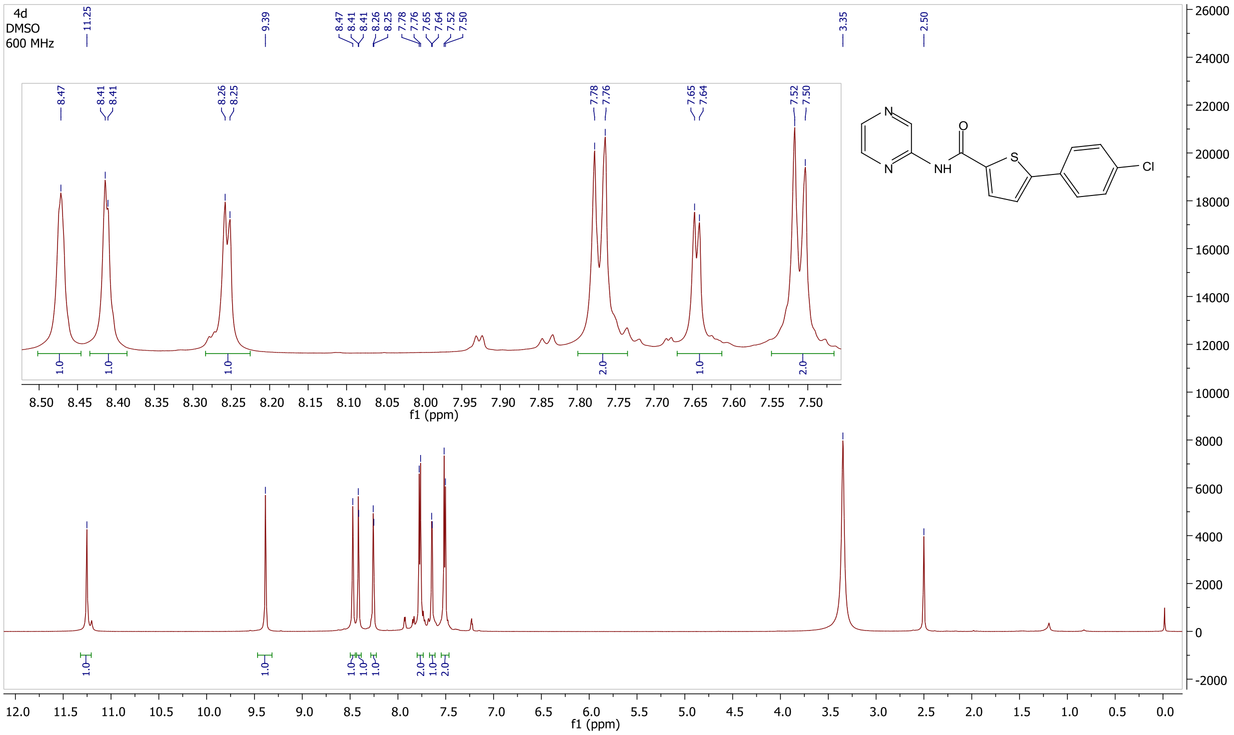

Figure S6: ^{1}H NMR (600 MHz, DMSO-d$_6$) of compound 4d.

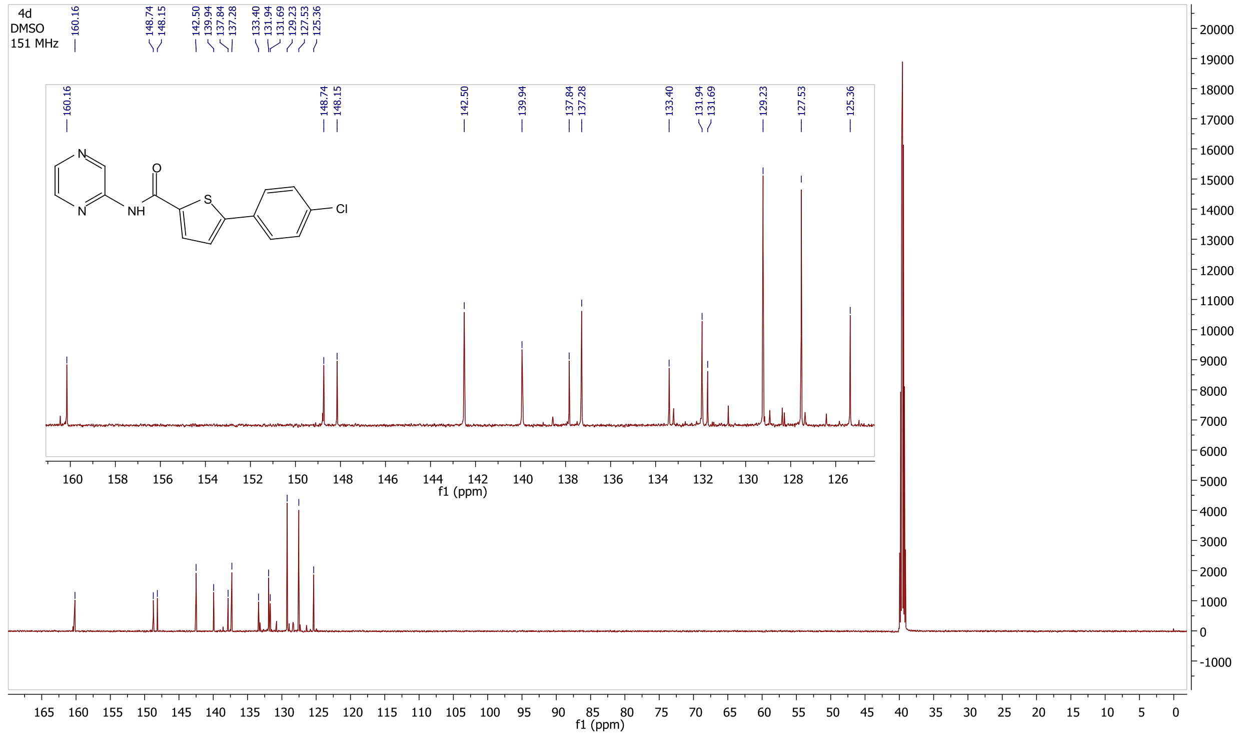

Figure S7: ^{13}C NMR (151 MHz, DMSO-d$_6$) of compound 4d.

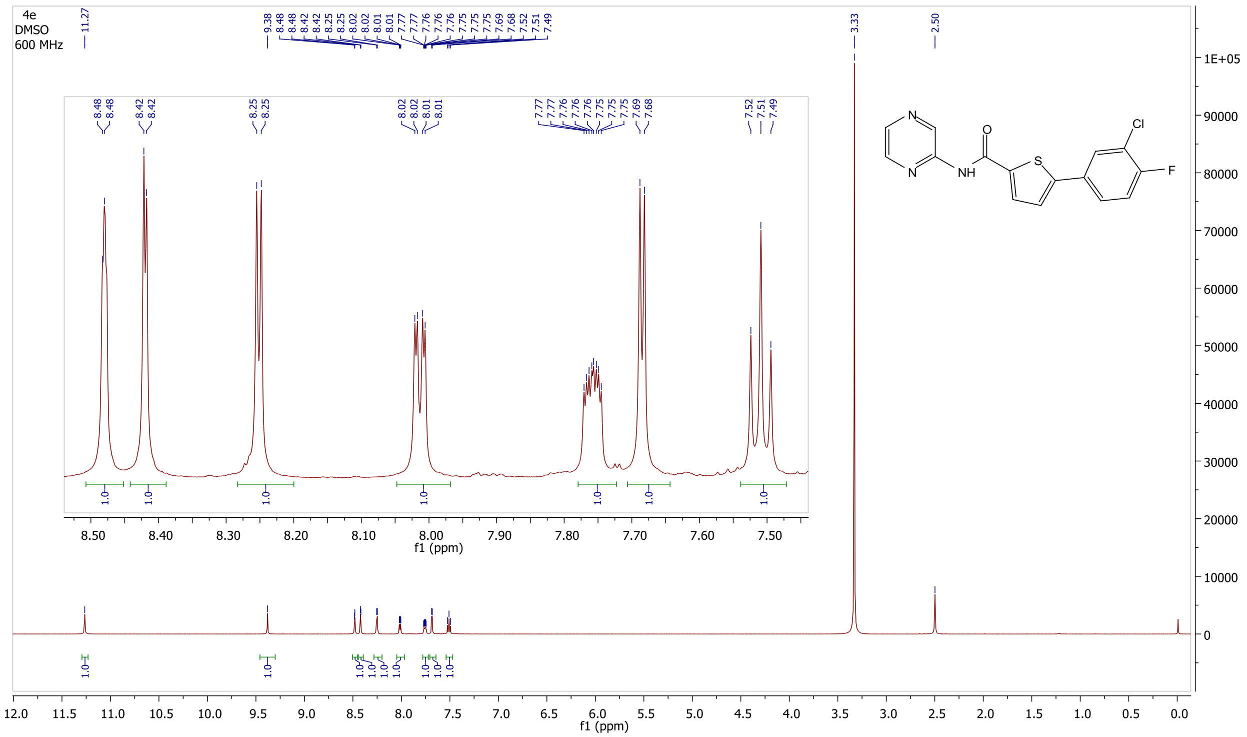

Figure S8: ¹H NMR (600 MHz, DMSO-d_6) of compound 4e.

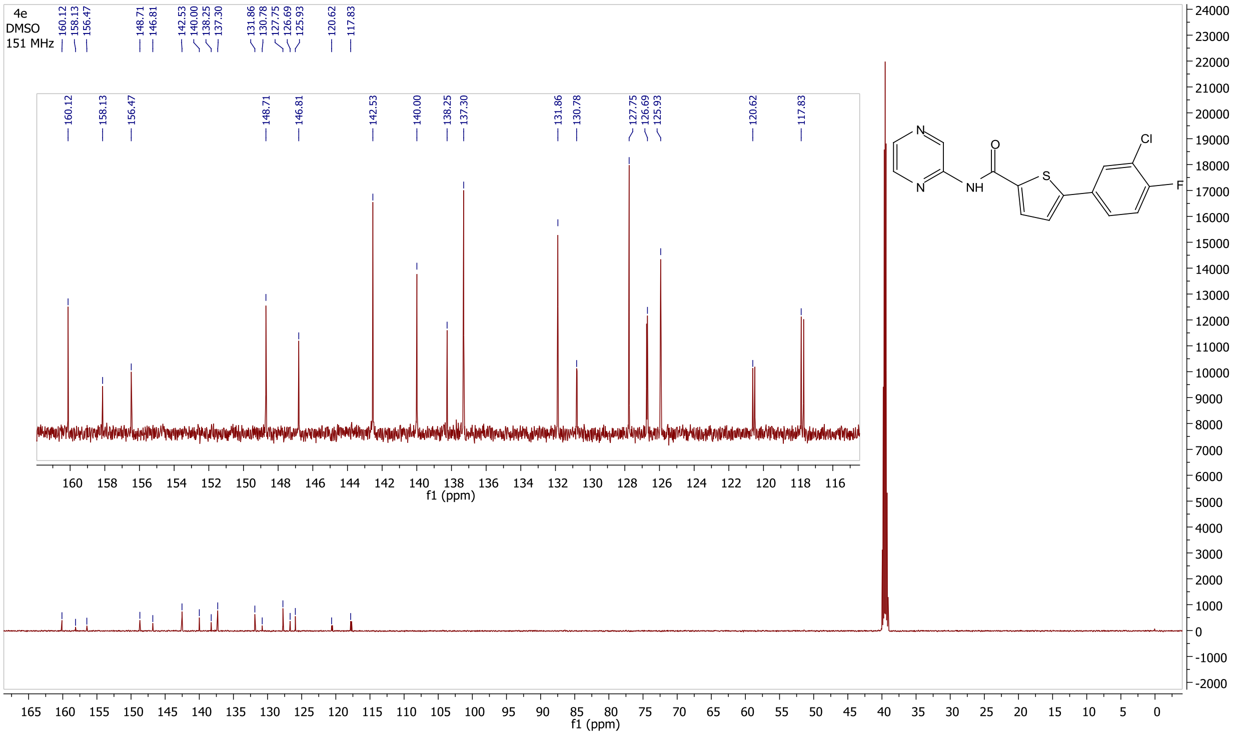

Figure S9: ^{13}C NMR (151 MHz, DMSO-d$_6$) of compound 4e.

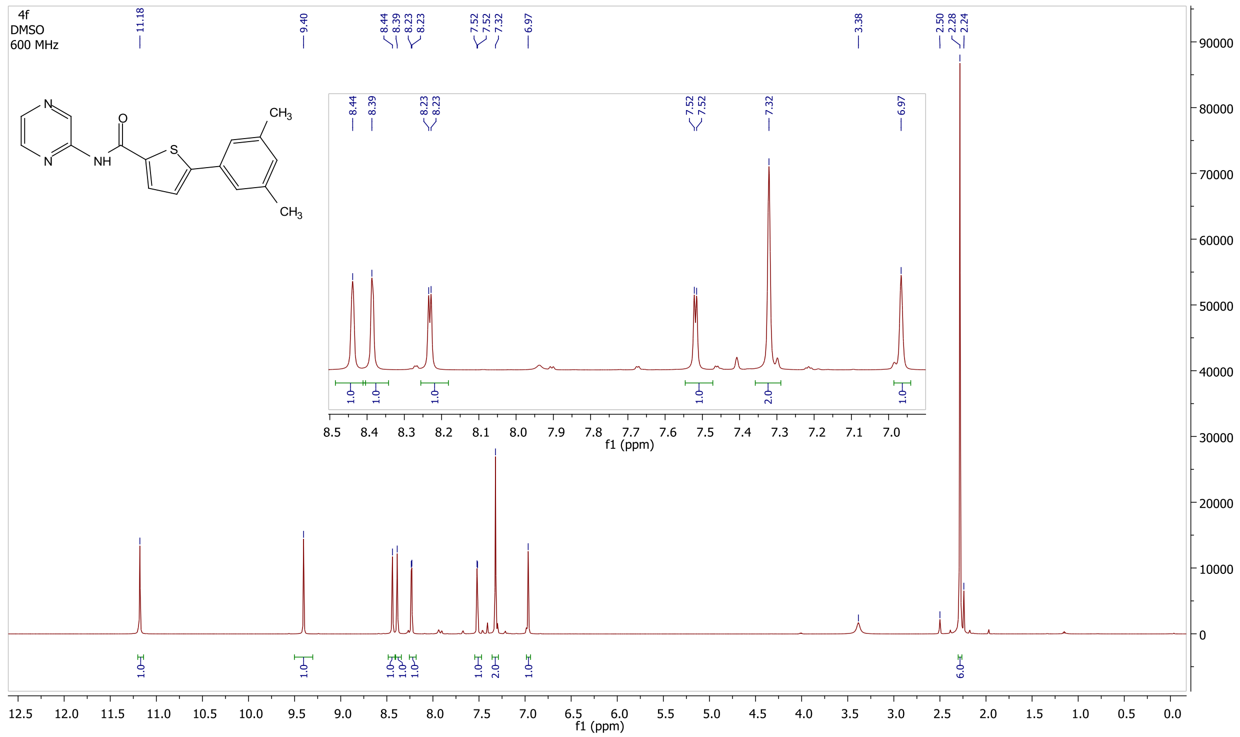

Figure S10: ^{1}H NMR (600 MHz, DMSO-d_6) of compound 4f.

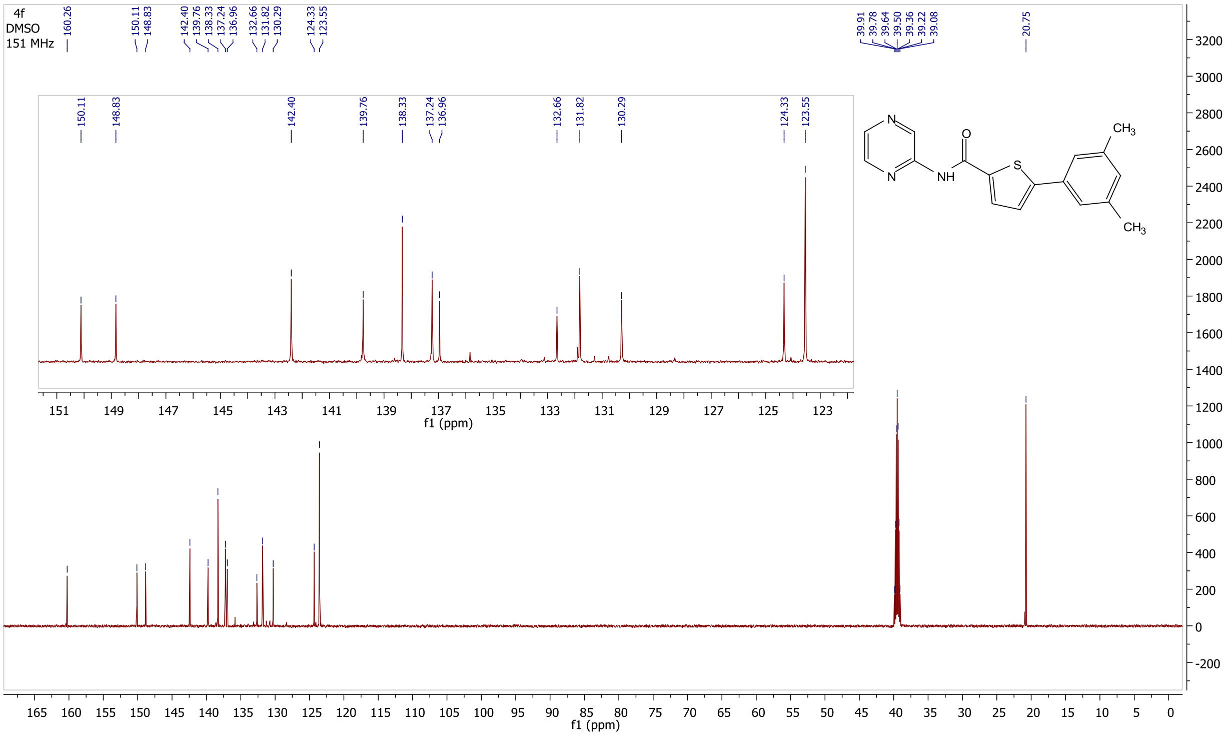

Figure S11: ^{13}C NMR (151 MHz, DMSO-d$_6$) of compound 4f.

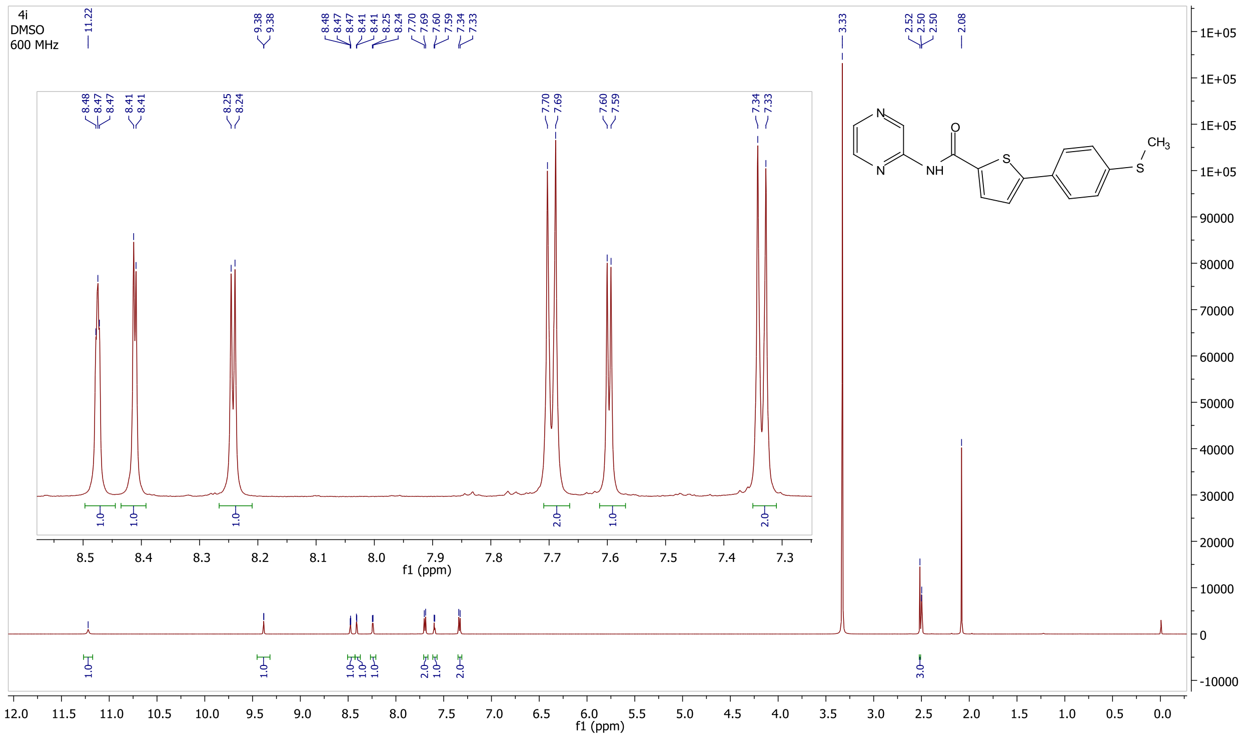

Figure S12: ^{1}H NMR (600 MHz, DMSO-d_6) of compound 4i.

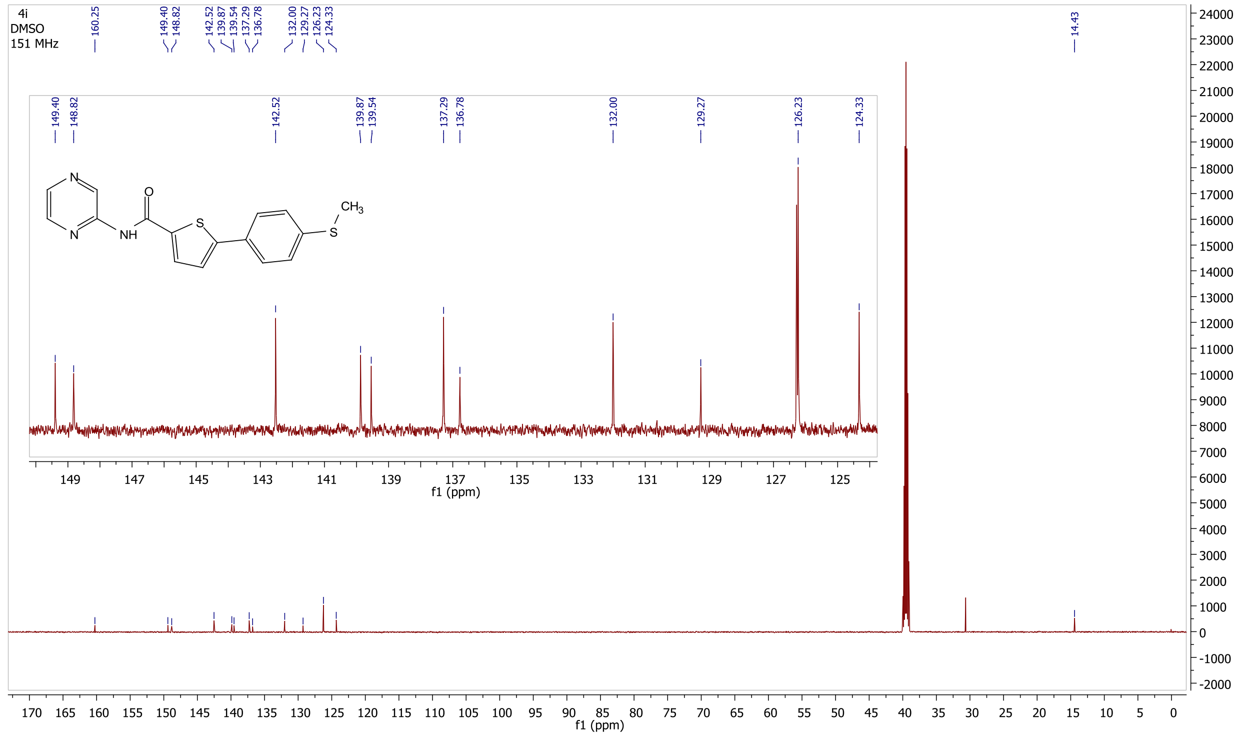

Figure S13: ^{13}C NMR (151 MHz, DMSO-d$_6$) of compound 4i.

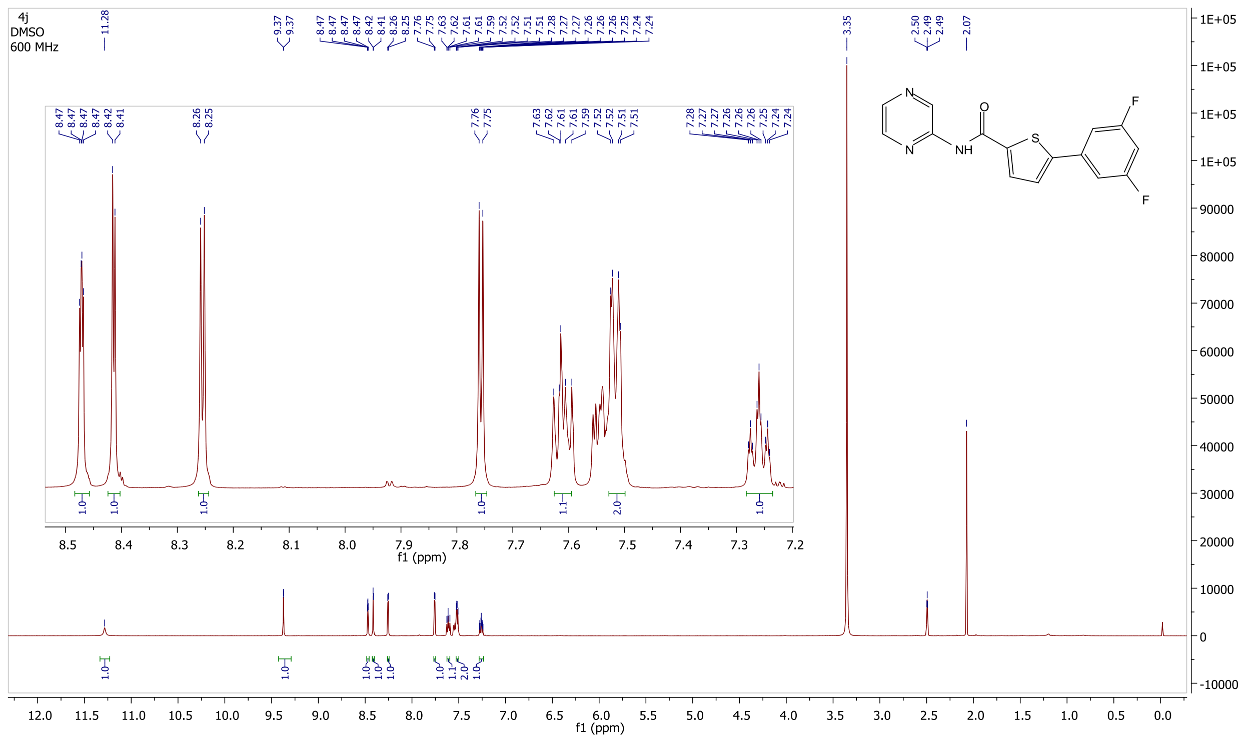

Figure S14: ^{1}H NMR (600 MHz, DMSO-d$_{6}$) of compound 4j.

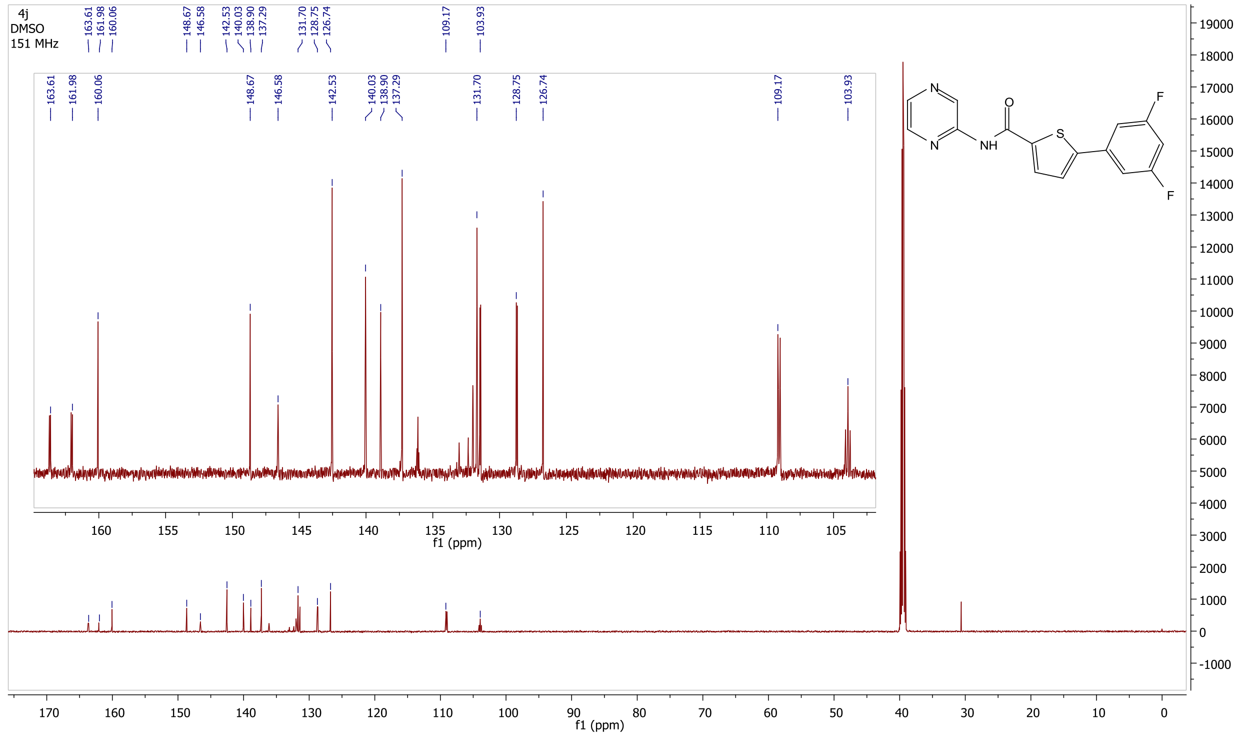

Figure S15: ^{13}C NMR (151 MHz, DMSO-d$_6$) of compound 4j.

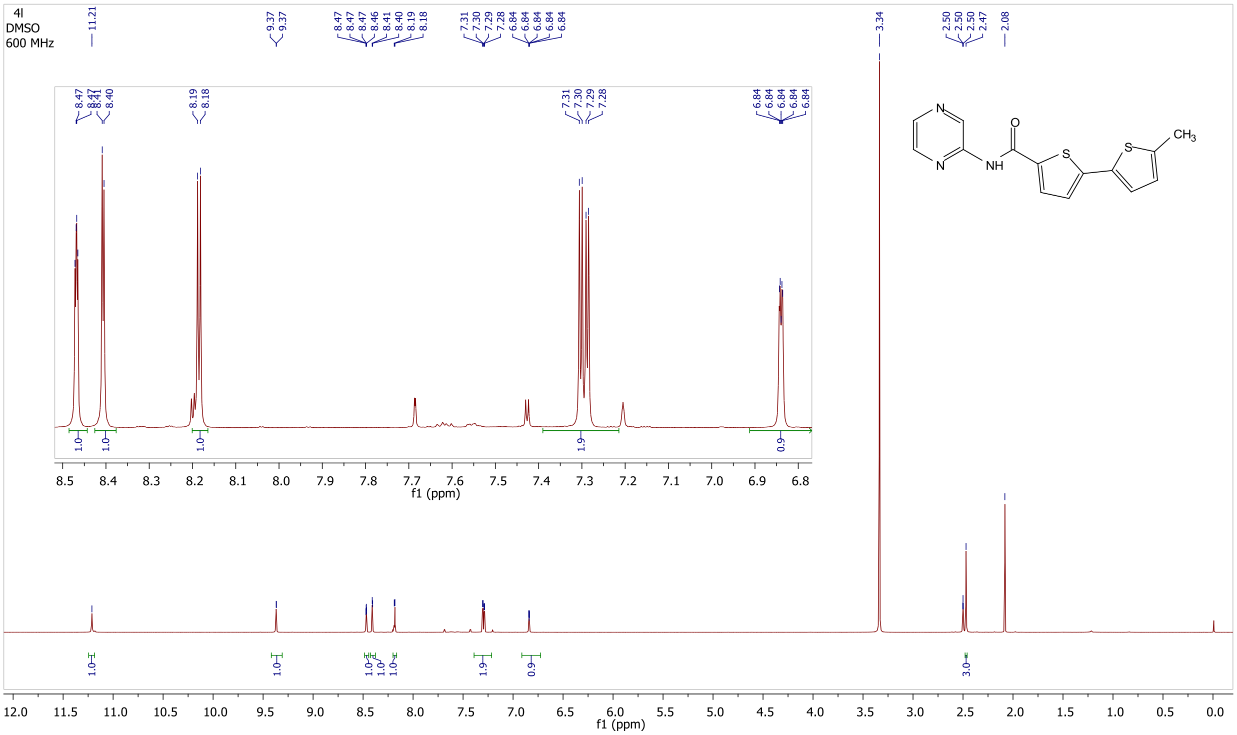

Figure S16: ^{1}H NMR (600 MHz, DMSO-d$_6$) of compound 4l.

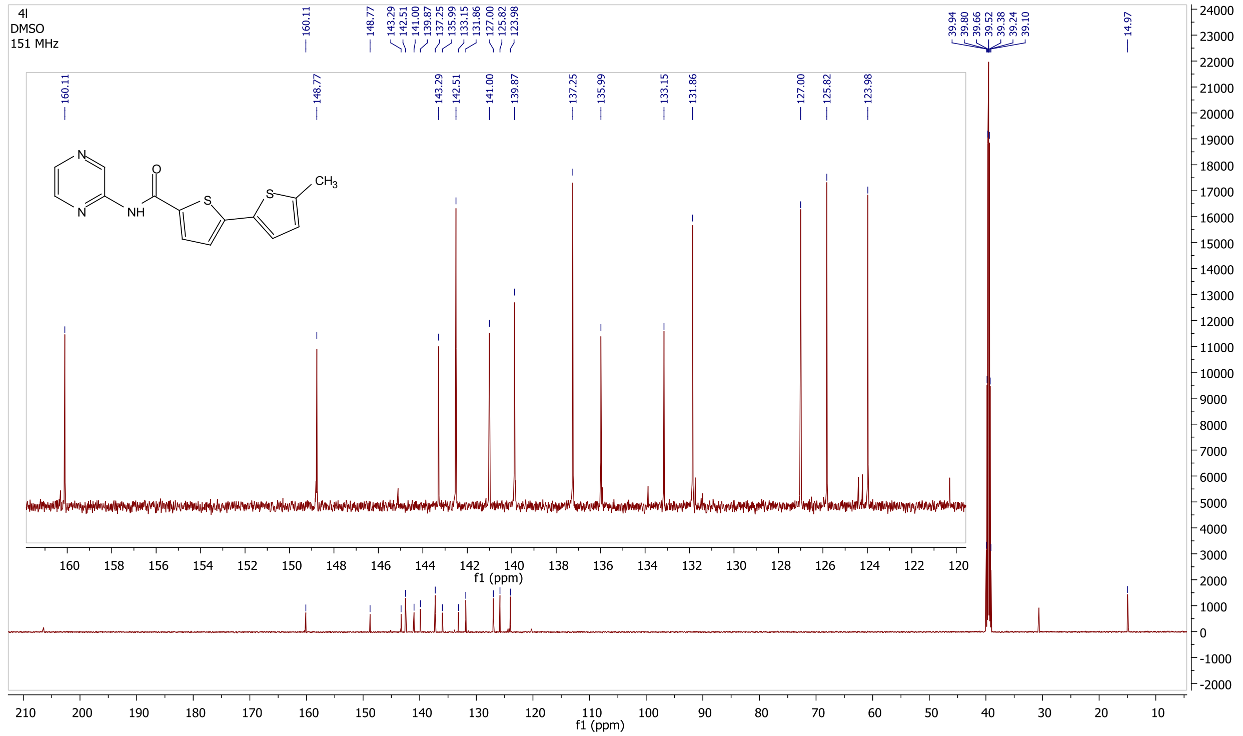

Figure S17: ^{13}C NMR (151 MHz, DMSO-d_6) of compound 4l.

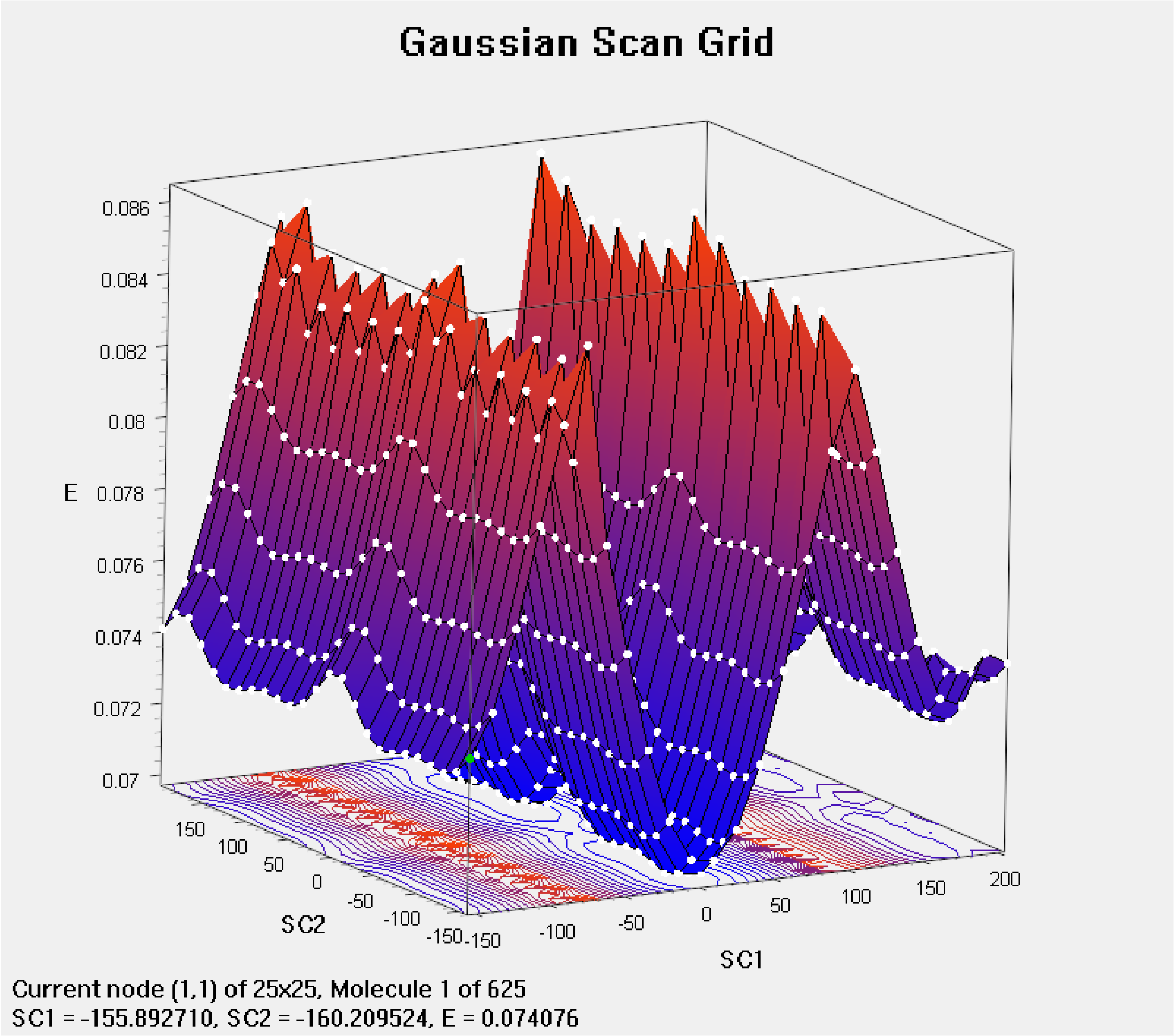

Figure S18. Potential Energy Scan of compound 4a at PBE0-D3BJ/def2-SVP/SMD$_{1,4\text{-dioxane}}$ level of theory

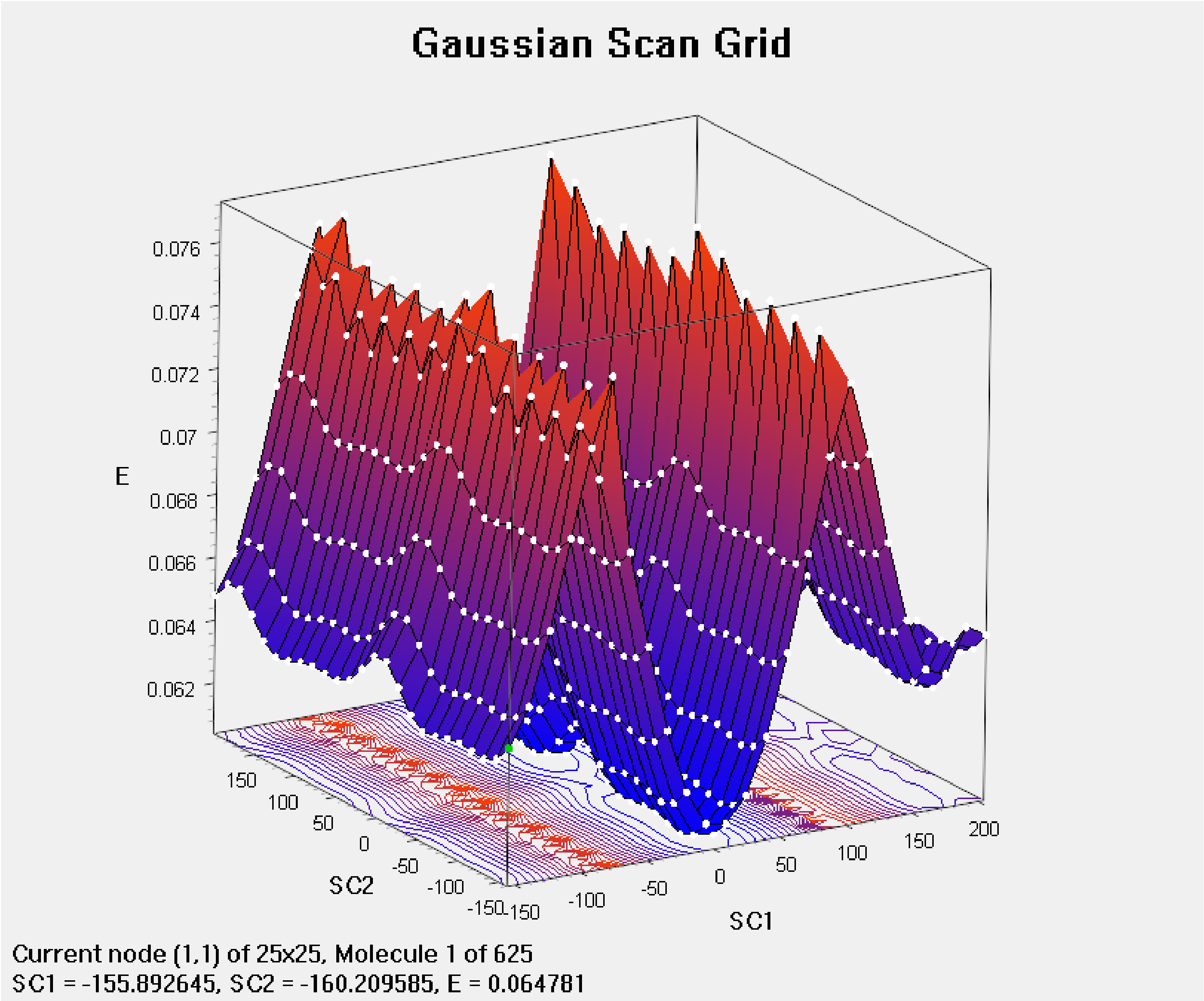

Figure S19. Potential Energy Scan of compound 4b at PBE0-D3BJ/def2-SVP/SMD$_{1,4\text{-dioxane}}$ level of theory

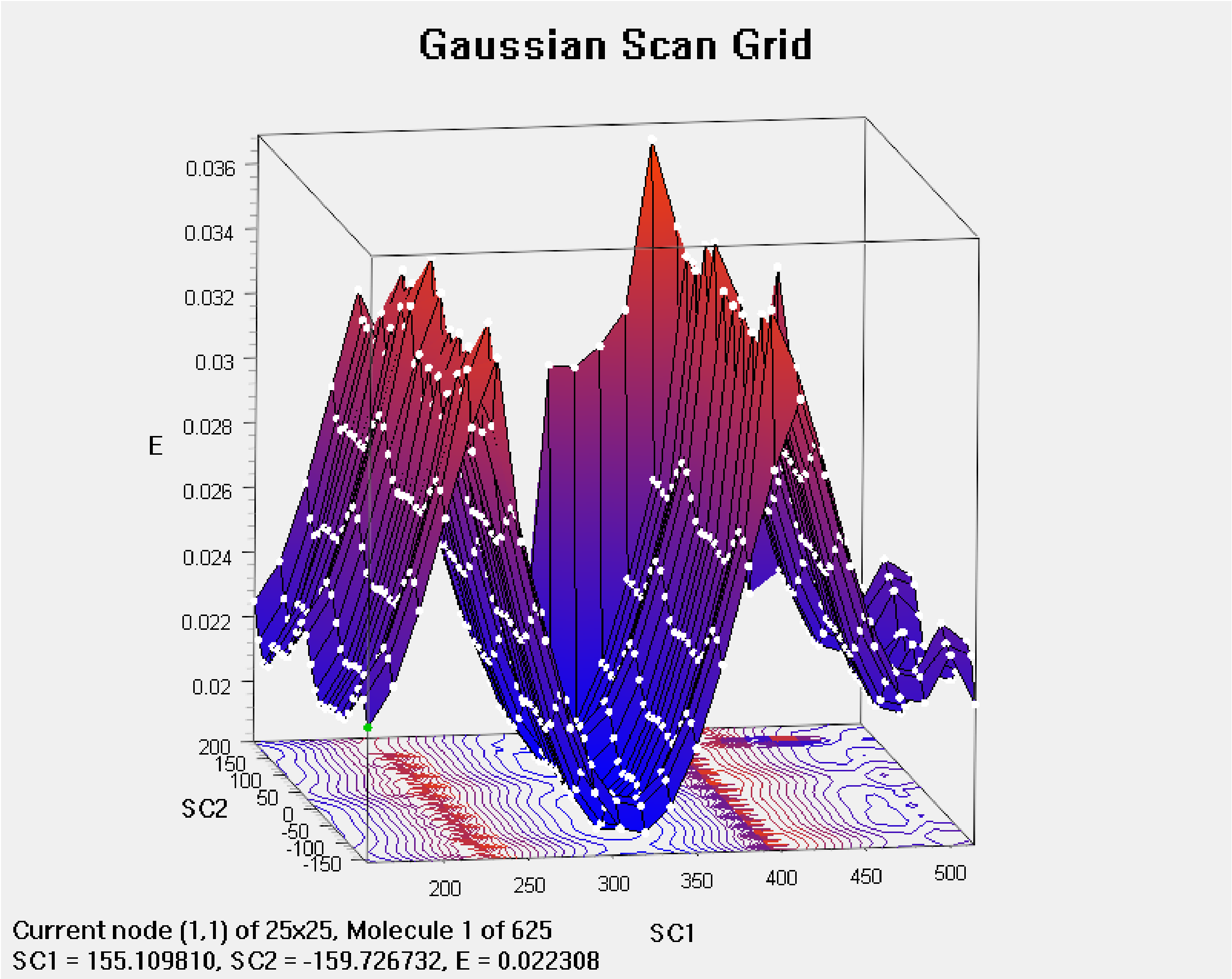

Figure S20. Potential Energy Scan of compound 4c at PBE0-D3BJ/def2-SVP/SMD$_{\text{1,4-dioxane}}$ level of theory

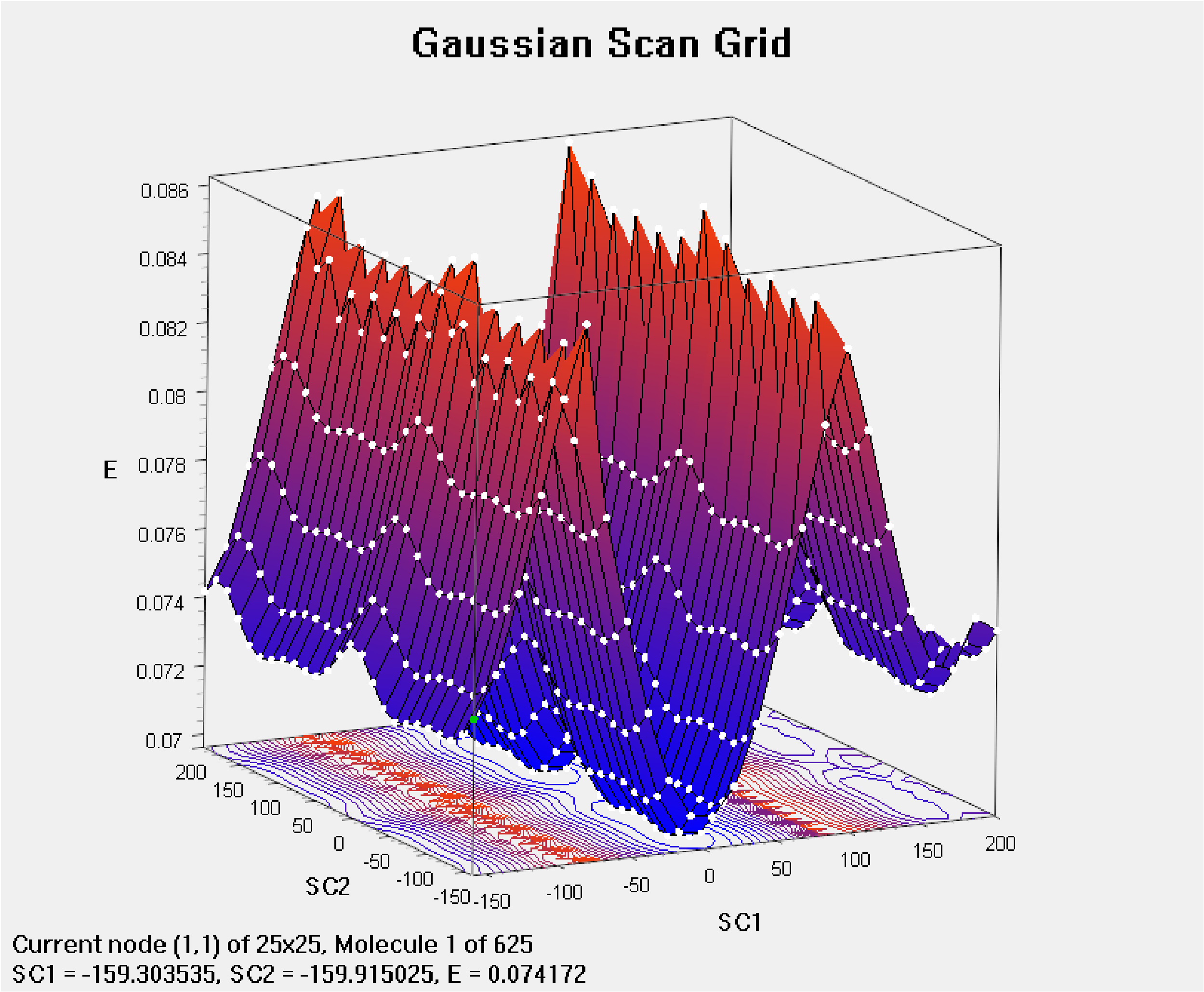

Figure S21. Potential Energy Scan of compound 4d at PBE0-D3BJ/def2-SVP/$SMD_{1,4\text{-dioxane}}$ level of theory

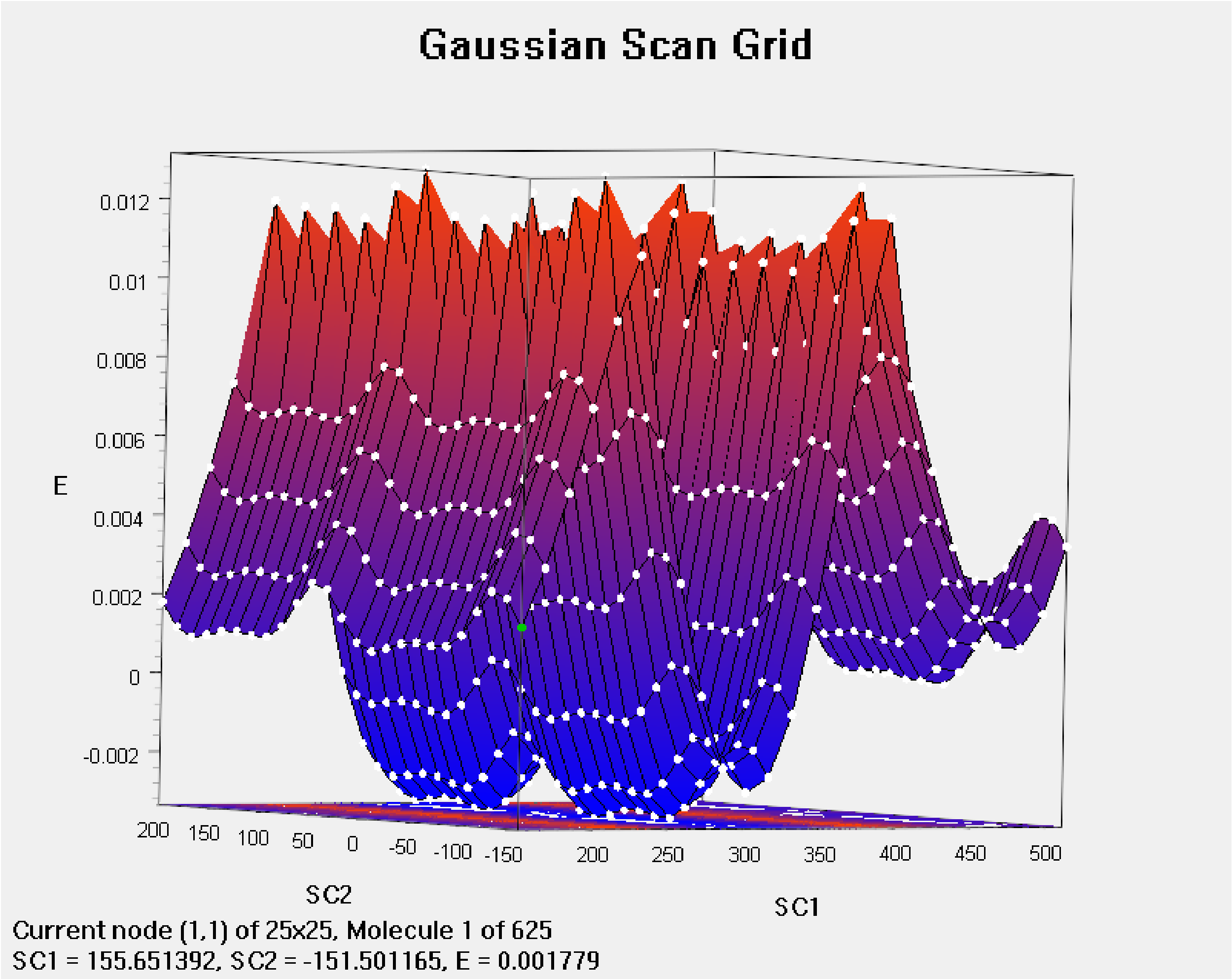

Figure S22. Potential Energy Scan of compound 4e at PBE0-D3BJ/def2-SVP/SMD$_{1,4\text{-dioxane}}$ level of theory

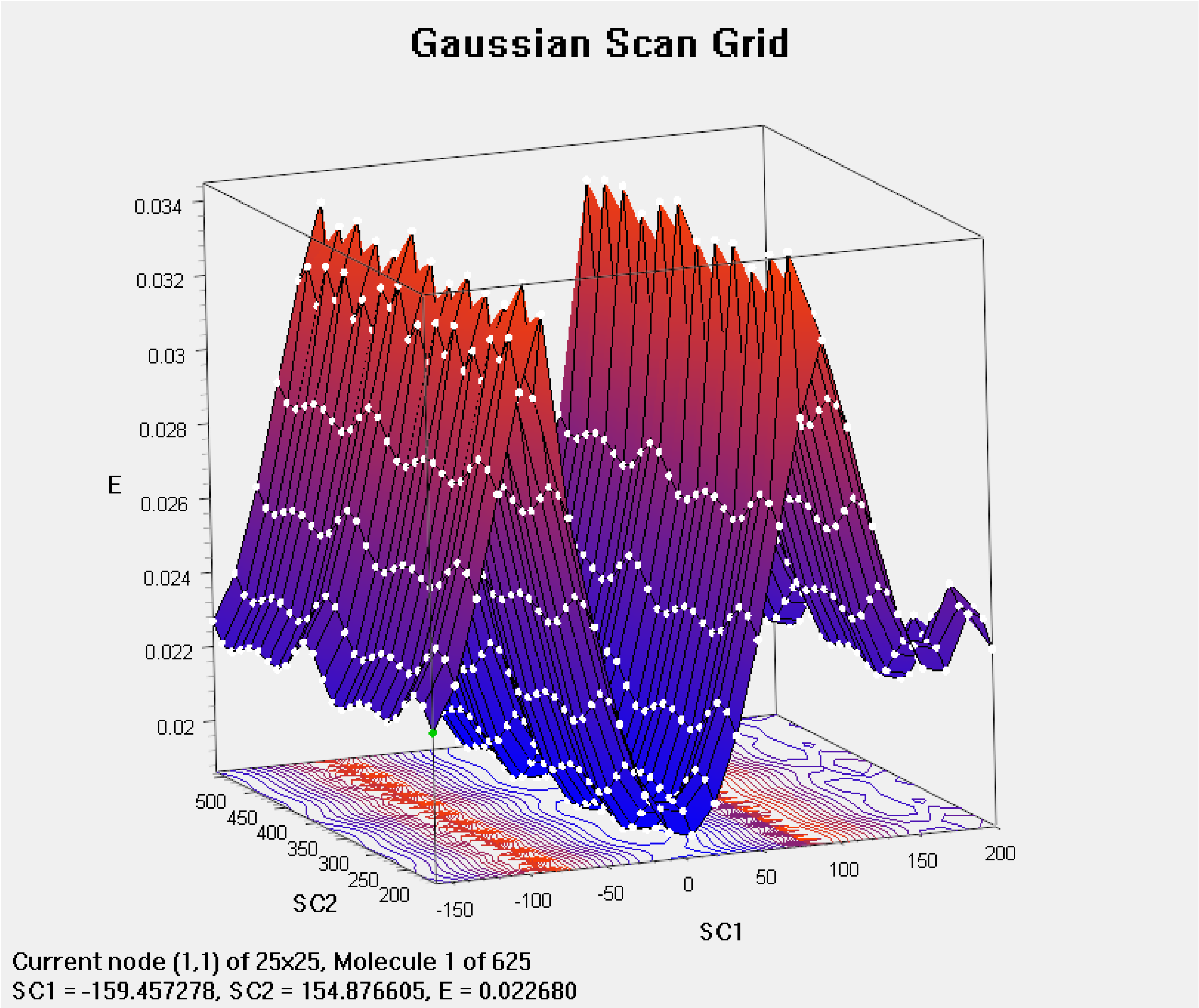

Figure S23. Potential Energy Scan of compound 4g at PBE0-D3BJ/def2-SVP/SMD$_{1,4\text{-dioxane}}$ level of theory

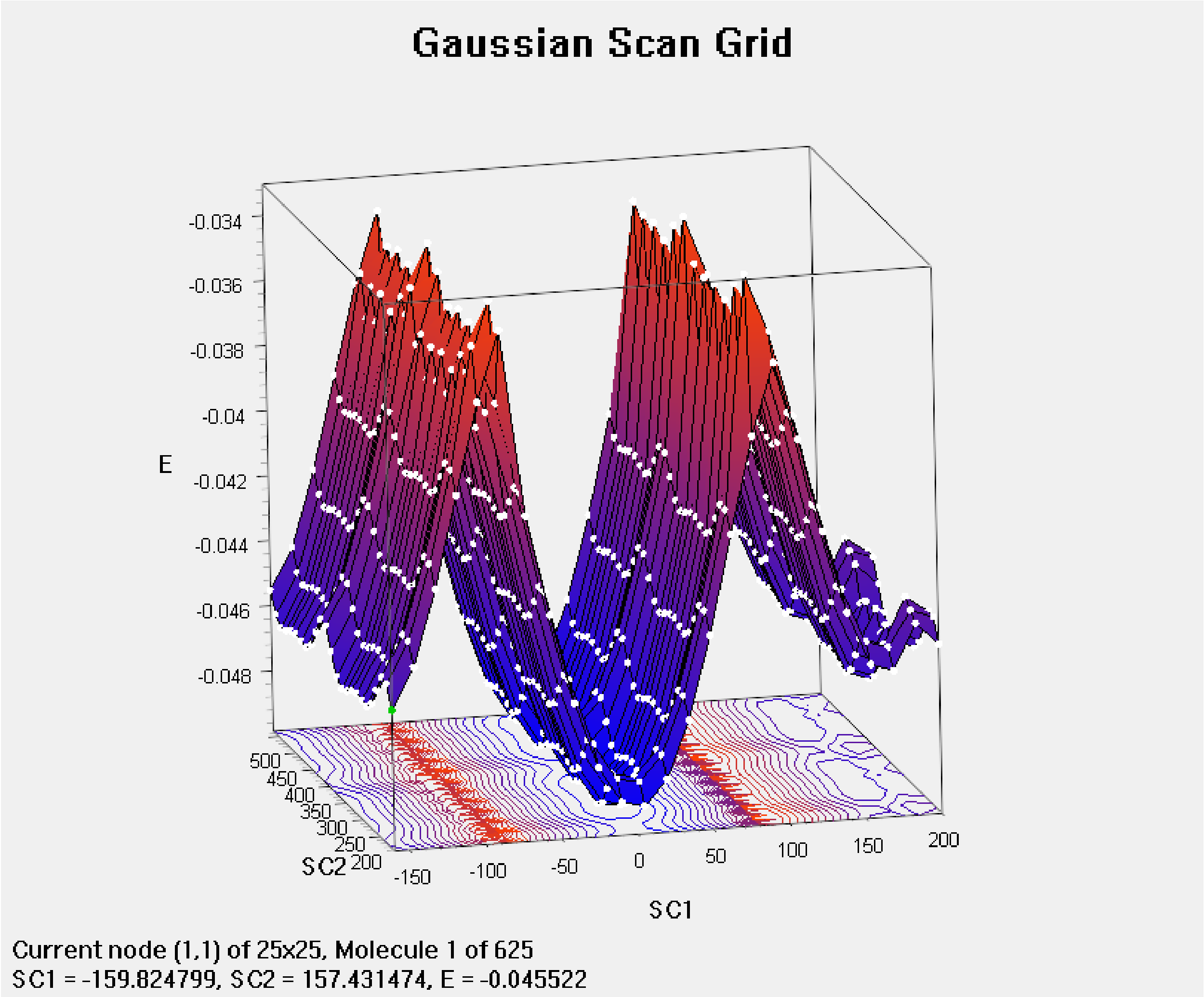

Figure S24. Potential Energy Scan of compound 4h at PBE0-D3BJ/def2-SVP/SMD$_{1,4\text{-dioxane}}$ level of theory

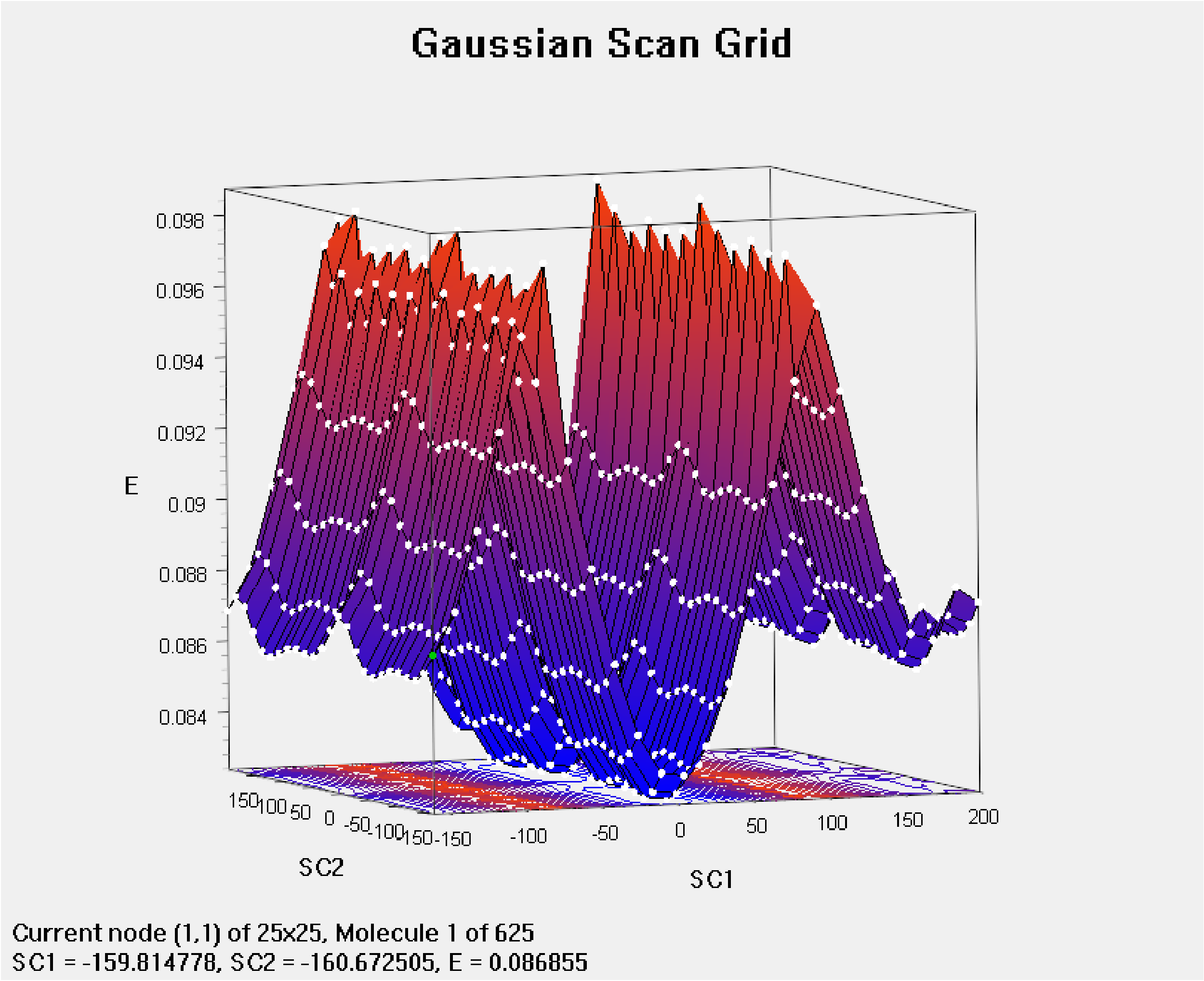

Figure S25. Potential Energy Scan of compound 4i at PBE0-D3BJ/def2-SVP/SMD$_{1,4\text{-dioxane}}$ level of theory

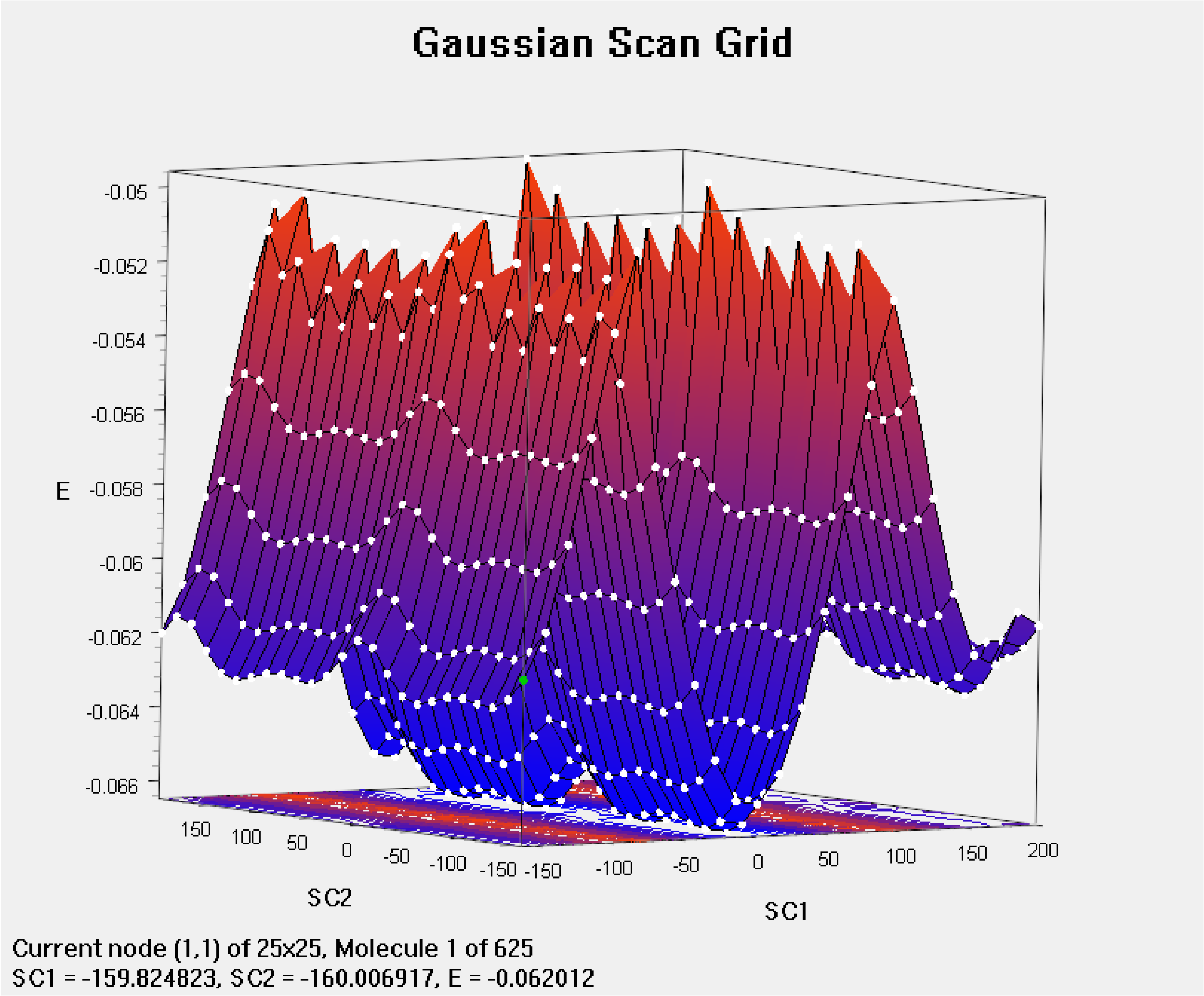

Figure S26. Potential Energy Scan of compound 4j at PBE0-D3BJ/def2-SVP/SMD$_{1,4\text{-dioxane}}$ level of theory

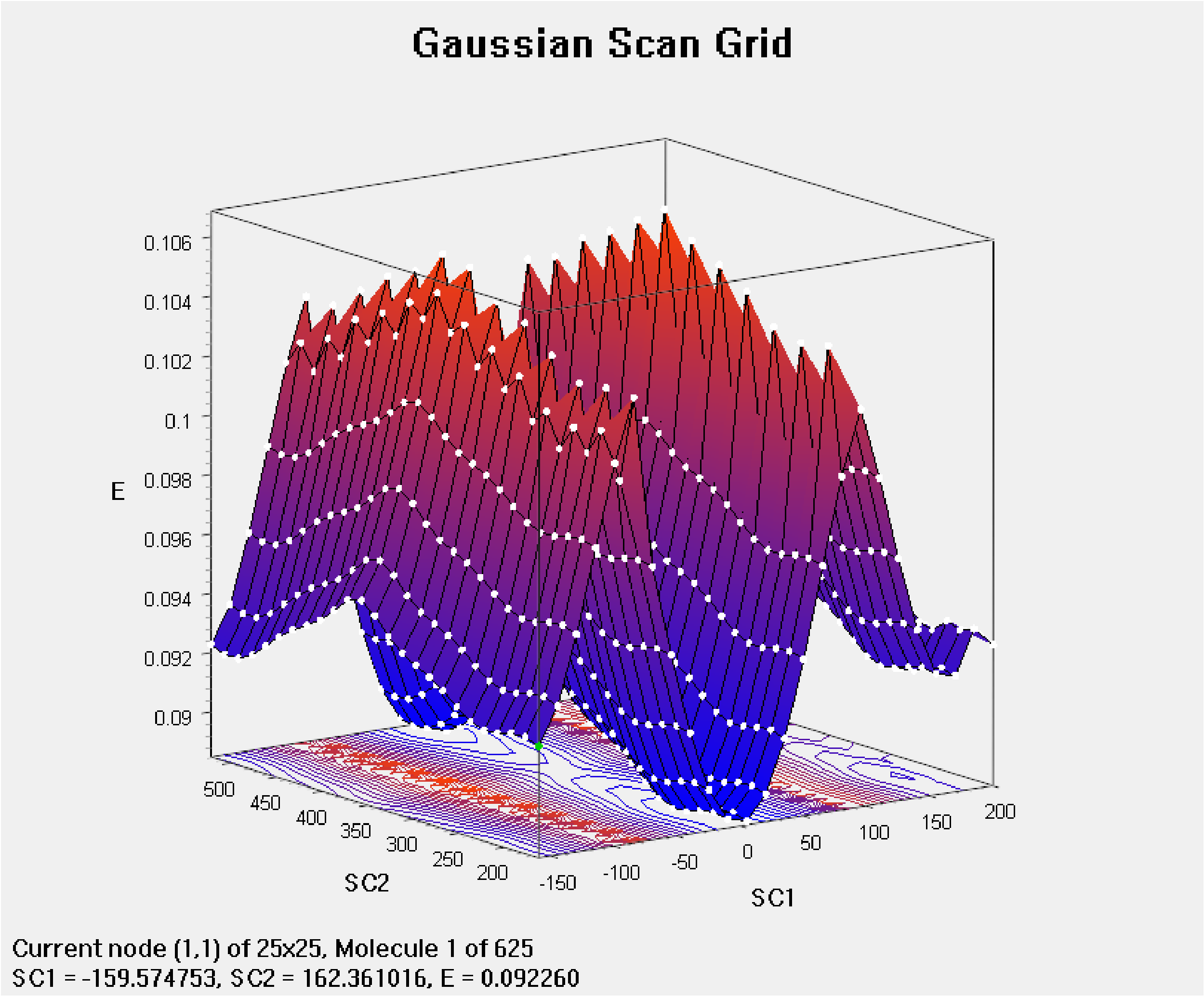

Figure S27. Potential Energy Scan of compound 4k at PBE0-D3BJ/def2-SVP/SMD$_{1,4\text{-dioxane}}$ level of theory

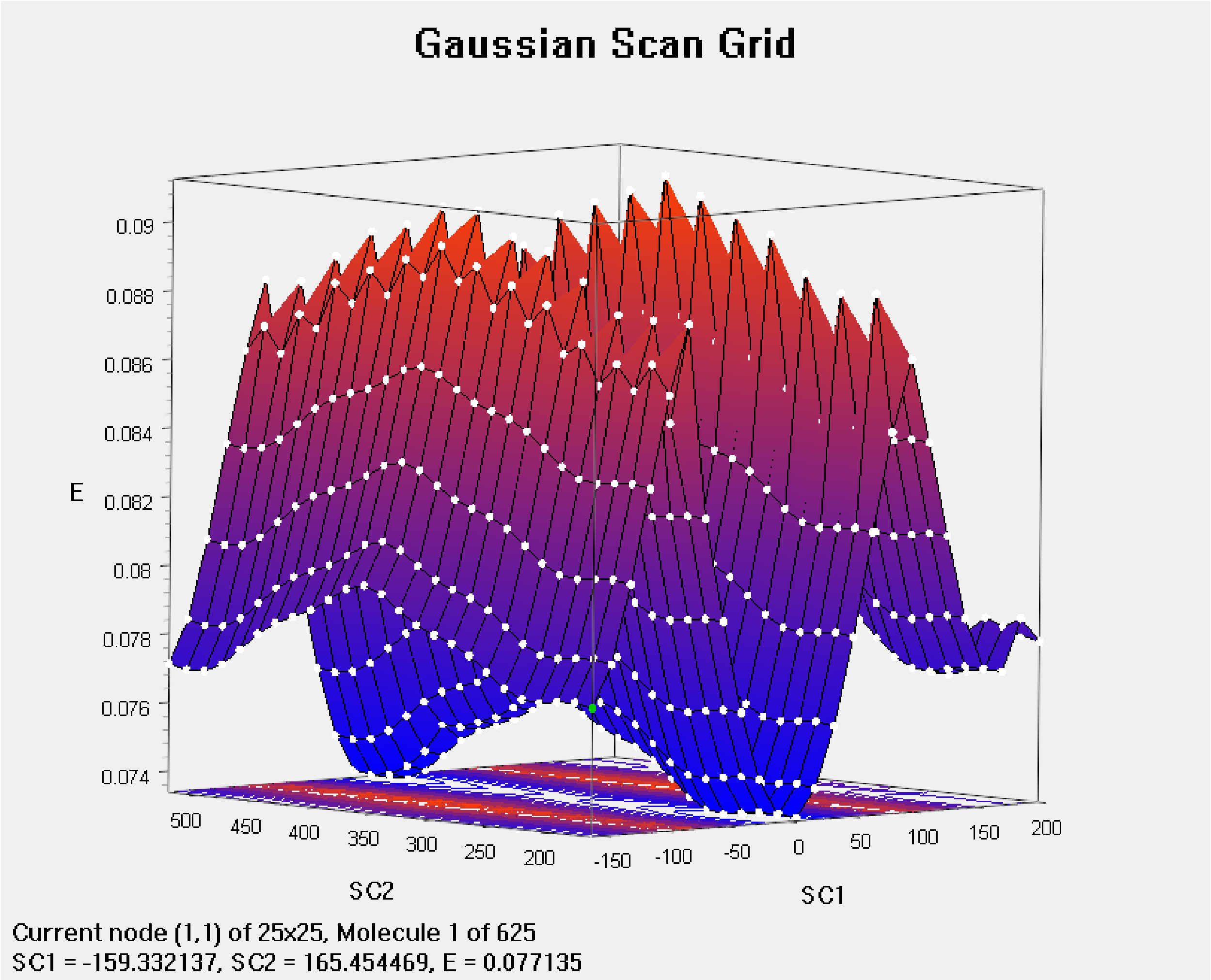

Figure S28. Potential Energy Scan of compound 4l at PBE0-D3BJ/def2-SVP/SMD$_{1,4\text{-dioxane}}$ level of theory

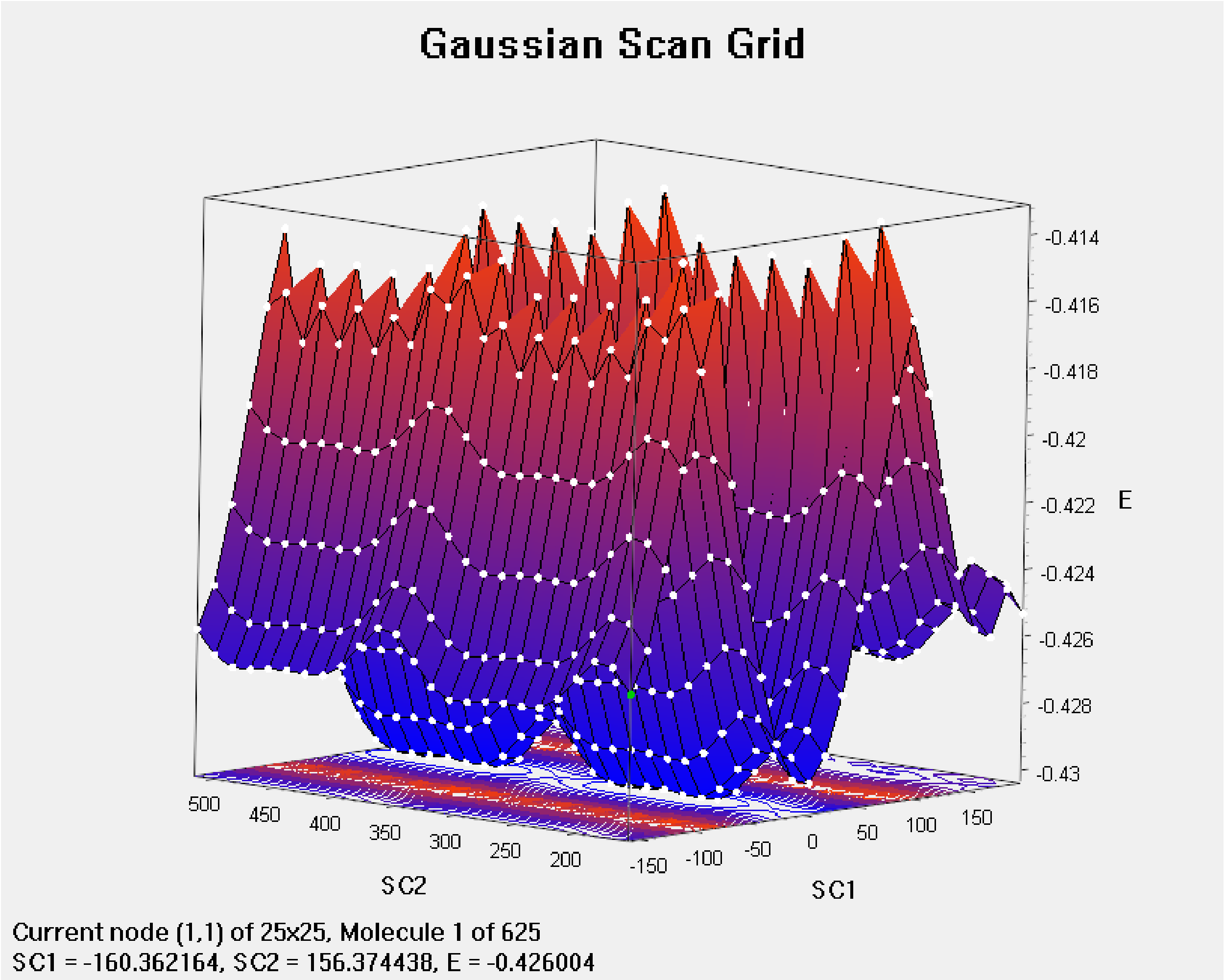

Figure S29. Potential Energy Scan of compound 4m at PBE0-D3BJ/def2-SVP/SMD$_{1,4\text{-dioxane}}$ level of theory

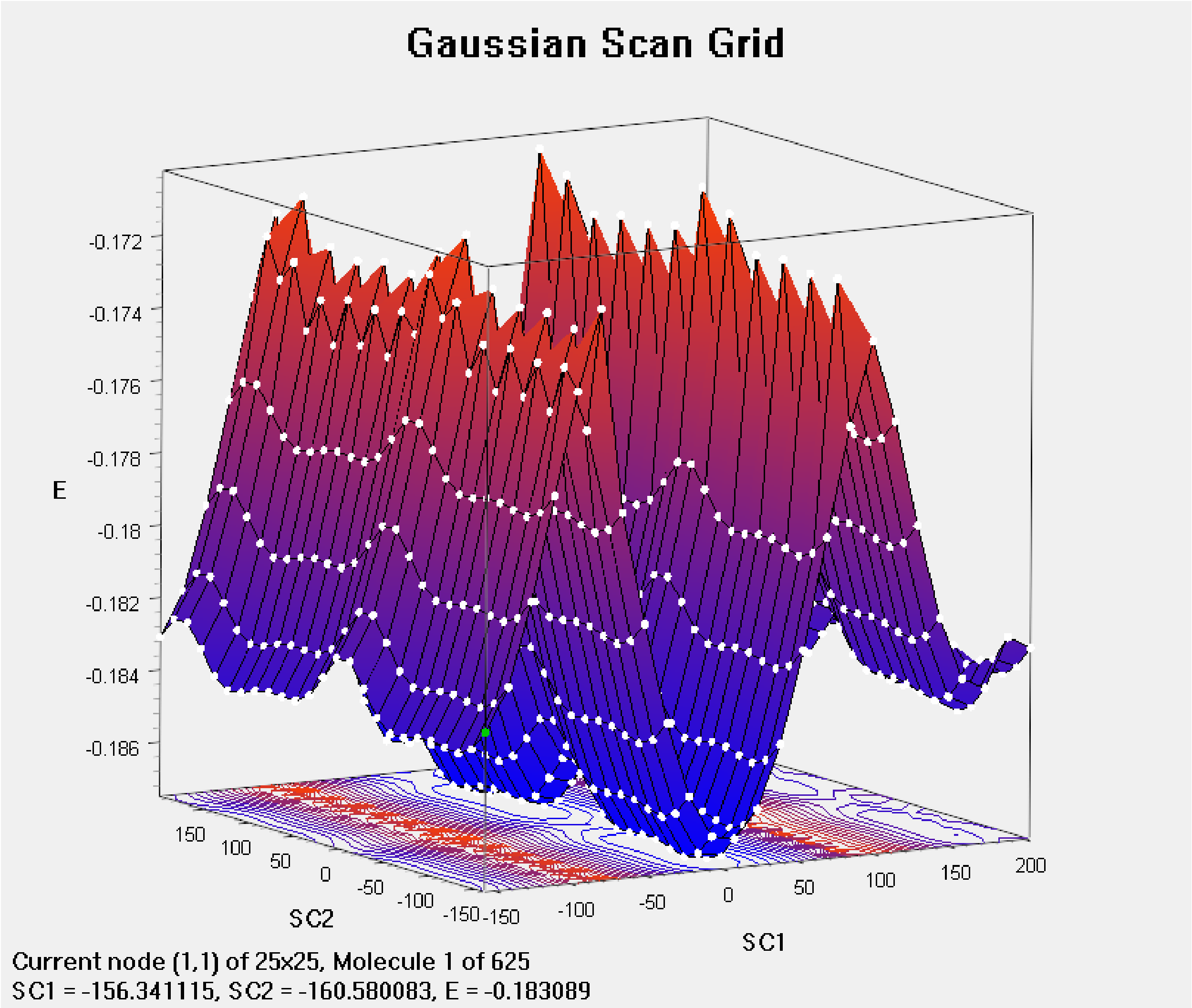

Figure S30. Potential Energy Scan of compound 4n at PBE0-D3BJ/def2-SVP/SMD$_{\text{1,4-dioxane}}$ level of theory

XYZ Coordinates of the lowest energy conformer of 4a

C	-7.28987900	0.01442100	0.31517900
C	-6.78901100	-0.95755400	-0.57535100
N	-5.45757800	-1.10483500	-0.78707600
C	-4.61991300	-0.26745800	-0.09564900
C	-5.13229400	0.71916400	0.80019700
N	-6.45852000	0.84724400	0.99605700
H	-8.36217500	0.13878300	0.49304800
H	-7.44806900	-1.63246900	-1.13320800
H	-4.46285400	1.40339200	1.35071400
N	-3.24068400	-0.48898700	-0.34170700
H	-3.03183600	-1.21801200	-1.03500200
C	-2.19033200	0.29879700	0.19372500
O	-2.40985200	1.27749600	0.88202500
C	-0.83961500	-0.16462100	-0.13169100
C	-0.34996500	-1.35729800	-0.60193200
S	0.44531100	0.95776200	0.15773300
C	1.07597600	-1.35589700	-0.74507300
H	-0.94647400	-2.22962000	-0.84026200
C	1.65260700	-0.16348700	-0.38201900
H	1.62218500	-2.21524800	-1.11295300
C	3.05666100	0.22262000	-0.38216500
C	4.00207600	-0.60673100	0.24690300
C	3.47107500	1.41368100	-1.00321000
C	5.34142400	-0.21802500	0.23900600
H	3.69136700	-1.52992400	0.73698100
C	4.82102100	1.77660300	-0.99440400
H	2.74325500	2.05433100	-1.50194600
C	5.77160400	0.96299200	-0.37130800
H	6.82495500	1.24258400	-0.36114400
H	5.13608100	2.70207100	-1.47806600
Cl	6.50566300	-1.23160300	1.00927900

XYZ Coordinates of the lowest energy conformer of 4b

C	-7.93083700	-0.05475000	-0.45525000
C	-7.40251400	1.20925700	-0.11988200
N	-6.07344400	1.39092500	0.07854400
C	-5.26613300	0.29112400	-0.06150500
C	-5.80520800	-0.98555900	-0.40360400
N	-7.12907100	-1.14347400	-0.59524600
H	-9.00184200	-0.21017600	-0.61636200
H	-8.03744300	2.09527300	-0.00556500
H	-5.15916700	-1.87327500	-0.52258700
N	-3.88962200	0.54604300	0.16871000
H	-3.65503500	1.52252800	0.38597900
C	-2.86150200	-0.42240200	0.06220500
O	-3.08879300	-1.56640700	-0.28305900
C	-1.51785600	0.06327200	0.38994100
C	-1.06744800	1.12599300	1.13157700
S	-0.19465200	-0.87654100	-0.20878500
C	0.36292200	1.18686900	1.20513000
H	-1.69608000	1.85227700	1.63265900
C	0.98070700	0.16601800	0.52549800
H	0.88172800	1.96026100	1.75742200
C	2.40014700	-0.10767500	0.35844200
C	3.25408200	0.91649600	-0.08838400
C	2.91977000	-1.38462500	0.63864200
C	4.61376400	0.64905300	-0.25106300
H	2.85730600	1.90851100	-0.31272600
C	4.28166600	-1.64387800	0.47332700
H	2.26178600	-2.17875800	0.99691600
C	5.12757700	-0.62437600	0.02719500
H	4.68572900	-2.63463400	0.69108600
Cl	5.66063100	1.89041300	-0.79578500
Cl	6.79807000	-0.93449900	-0.17830100

XYZ Coordinates of the lowest energy conformer of 4c

C	-7.37184300	0.78869800	-0.06218700
C	-7.03238100	-0.36360700	0.67660600
N	-5.74512400	-0.77230100	0.79918000
C	-4.78834800	-0.01571200	0.17209200
C	-5.13677200	1.15178300	-0.57202700
N	-6.42203100	1.53913200	-0.68096000
H	-8.40771800	1.12490200	-0.16678300
H	-7.78776700	-0.97621600	1.18181100
H	-4.36962300	1.76764400	-1.07395600
N	-3.46569200	-0.50398100	0.32771800
H	-3.37687700	-1.34987400	0.90343600
C	-2.30731500	0.12792000	-0.18807400
O	-2.36970700	1.17695300	-0.80265300
C	-1.04558600	-0.57237100	0.05773100
C	-0.72950800	-1.77252100	0.64247500
S	0.38204800	0.23450300	-0.50093300
C	0.67905700	-2.03742800	0.64756400
H	-1.44080600	-2.47156000	1.06526900
C	1.41868100	-1.03641000	0.06761200
H	1.09660000	-2.93656700	1.08206800
C	2.85740700	-0.93368500	-0.12072700
C	3.56832100	-2.00932300	-0.68495800
C	3.54169500	0.23336000	0.25762800
C	4.94914700	-1.91619700	-0.86491700
H	3.03642500	-2.91270200	-0.98518500
C	4.92510600	0.32244500	0.06993200
H	2.99427500	1.06068100	0.71151600
C	5.62991900	-0.75639400	-0.48448300
H	5.49913100	-2.75092300	-1.29958200
H	6.71432400	-0.68368200	-0.60831000
C	5.70009800	1.54673600	0.46575700
O	6.86247300	1.46701700	0.79799600
C	4.98081100	2.86182400	0.40691800
H	4.39148800	2.97987400	-0.51136400
H	4.30024100	2.98961700	1.26003700
H	5.69441300	3.70035100	0.45001700

XYZ Coordinates of the lowest energy conformer of 4d

C	-7.38336100	-0.41920600	0.01334300
C	-6.92486000	0.79754000	0.55908800
N	-5.60253100	1.09247900	0.62045400
C	-4.73111700	0.15526200	0.12722300
C	-5.20015200	-1.07779100	-0.41891100
N	-6.51811800	-1.34940100	-0.47094400
H	-8.44816000	-0.66432800	-0.04145200
H	-7.61111100	1.55413600	0.95616600
H	-4.50231600	-1.84043900	-0.80723900
N	-3.36608500	0.53357300	0.19823300
H	-3.18631400	1.44098900	0.64554000
C	-2.27916800	-0.29508900	-0.18046600
O	-2.44670500	-1.43968500	-0.55619300
C	-0.96107600	0.33736300	-0.09478500
C	-0.55855900	1.64810100	-0.04340800
S	0.40438000	-0.72635800	-0.10689700
C	0.86665800	1.79189400	0.00542300
H	-1.21891900	2.50689400	-0.04757300
C	1.53056000	0.59012300	-0.01019600
H	1.34946500	2.75907800	0.05937800
C	2.96012900	0.32114100	0.02177900
C	3.50007500	-0.55098000	0.98390400
C	3.80557500	0.93978000	-0.91814400
C	4.87254000	-0.80662500	1.00785000
H	2.85122200	-1.02549900	1.72174900
C	5.17808800	0.68817800	-0.90203000
H	3.38495500	1.61287200	-1.66622400
C	5.69089100	-0.18298800	0.06288100
H	5.29752900	-1.48130500	1.75178800

XYZ Coordinates of the lowest energy conformer of 4e

C	7.59458400	0.01418600	-0.44930700
C	7.06129800	1.25243200	-0.03510100
N	5.73150500	1.41602000	0.17405600
C	4.92846500	0.32415400	-0.03555400
C	5.47281800	-0.92682500	-0.45554600
N	6.79721800	-1.06702100	-0.65675400
H	8.66611800	-0.12645500	-0.62010000
H	7.69274200	2.13190700	0.13519600
H	4.83048900	-1.80862700	-0.62748400
N	3.55019200	0.56081900	0.20317300
H	3.31316800	1.51894400	0.48808900
C	2.52797200	-0.40798000	0.05392000
O	2.76535000	-1.53908300	-0.32645700
C	1.17642900	0.05857100	0.37483100
C	0.70136600	1.12751400	1.09164600
S	-0.12584800	-0.92037900	-0.20898000
C	-0.73003200	1.15973100	1.16112200
H	1.31238200	1.87912100	1.57674000
C	-1.32486800	0.11165500	0.50275700
H	-1.26595700	1.93367800	1.69578200
C	-2.73716600	-0.19579400	0.33803700
C	-3.22776500	-1.47865000	0.64289900
C	-3.61080400	0.80536400	-0.12812600
C	-4.58328900	-1.77886600	0.48467500
H	-2.54998500	-2.24972300	1.01599800
C	-4.96330300	0.50957100	-0.28720500
H	-3.23264400	1.80079700	-0.36958400
C	-5.43424700	-0.77544300	0.01905800
H	-4.96363700	-2.77371400	0.72112700
F	-6.72991600	-1.02257900	-0.14446500
Cl	-6.05904800	1.69232700	-0.85130600

XYZ Coordinates of the lowest energy conformer of 4f

C	-7.24929900	0.29340200	0.03011500
C	-6.77378100	-0.98958100	0.36967300
N	-5.44673600	-1.26878700	0.39878900
C	-4.58742200	-0.24735900	0.08287400
C	-5.07469200	1.05145800	-0.25776300
N	-6.39643300	1.30596500	-0.28098800
H	-8.31757900	0.52669200	0.00352500
H	-7.44967500	-1.81373500	0.62403600
H	-4.38658200	1.87842600	-0.50773100
N	-3.21669400	-0.60516000	0.12063900
H	-3.02433700	-1.57445600	0.40095000
C	-2.14092500	0.28354700	-0.13798600
O	-2.33165300	1.45989300	-0.38417400
C	-0.80967200	-0.31957000	-0.08371000
C	-0.36842800	-1.61995900	-0.07483400
S	0.52581400	0.77747000	-0.05848700
C	1.05742300	-1.72288500	-0.03455300
H	-1.00340000	-2.49680100	-0.10342700
C	1.69758100	-0.50340700	-0.01299800
H	1.56212900	-2.68025500	-0.01921500
C	3.12386400	-0.20846700	0.01920800
C	4.05146100	-1.25894300	-0.09396600
C	3.58386100	1.11197200	0.15237100
C	5.42768000	-0.99316600	-0.07271200
H	3.70293400	-2.28326700	-0.20185600
C	4.96083900	1.37963400	0.17276000
H	2.88761300	1.94322200	0.24630600
C	5.88019400	0.32709300	0.06051000
C	5.45363800	2.78338900	0.31379300
H	6.07560600	2.90010800	1.21324200
H	6.06927800	3.07907000	-0.54829300
H	4.63975600	3.51643200	0.39090100
C	6.41506500	-2.10860500	-0.19262500
H	7.05709500	-1.97919700	-1.07628200
H	7.07594600	-2.15284700	0.68534600
H	5.93873300	-3.09310200	-0.28475500
H	6.94740800	0.53560700	0.07640800

XYZ Coordinates of the lowest energy conformer of 4g

C	7.38318200	-0.43146000	-0.22236700
C	6.93254400	0.90342500	-0.27027600
N	5.61291600	1.20910600	-0.19876400
C	4.73574400	0.16159600	-0.07705200
C	5.19772700	-1.18952300	-0.03481300
N	6.51248000	-1.46970500	-0.10602400
H	8.44534400	-0.68641300	-0.27780700
H	7.62276600	1.74894000	-0.36743600
H	4.49487100	-2.03676600	0.05223800
N	3.37508800	0.54613000	0.01553900
H	3.19855600	1.55551500	-0.06119700
C	2.28121500	-0.35769100	0.08392600
O	2.44362500	-1.56062100	0.01098800
C	0.97451000	0.27655200	0.25436100
C	0.59310700	1.49315900	0.76132900
S	-0.40983600	-0.65872400	-0.20294000
C	-0.82804600	1.67467900	0.77285000
H	1.26817300	2.25258200	1.13715400
C	-1.51692600	0.59346900	0.28089500
H	-1.29229000	2.57551400	1.15157400
C	-2.94377900	0.39224700	0.11537400
C	-3.56273900	-0.77948000	0.57905000
C	-3.72107100	1.38753100	-0.51946400
C	-4.93608800	-0.97421000	0.41709300
H	-2.97263800	-1.54871700	1.08162300
C	-5.08525600	1.21356900	-0.69257900
H	-3.23796600	2.29655800	-0.88154700
C	-5.68443900	0.02444000	-0.21925600
H	-5.70237000	1.96447200	-1.18195500
H	-5.39797800	-1.88518200	0.78319200
O	-7.03630000	-0.01444300	-0.45577700
C	-7.77196600	-1.18648500	-0.02539700
H	-7.71695500	-1.29260100	1.06171800
H	-8.79289800	-0.93635400	-0.34423200
H	-7.40753300	-2.07708400	-0.54528600

XYZ Coordinates of the lowest energy conformer of 4h

C	8.06698400	-0.51469800	-0.30128900
C	7.63955800	0.82911300	-0.27783600
N	6.32679500	1.15275600	-0.17351200
C	5.43356800	0.11535000	-0.09107300
C	5.87066600	-1.24331100	-0.12117700
N	7.17998800	-1.54194000	-0.22398500
H	9.12410300	-0.78450900	-0.38360700
H	8.34366100	1.66646500	-0.34309700
H	5.15502900	-2.08240300	-0.06621300
N	4.08021800	0.52013100	0.03980500
H	3.92232400	1.53538100	0.01430100
C	2.97403900	-0.36565800	0.07304900
O	3.10758600	-1.56645400	-0.06435000
C	1.67695400	0.28004200	0.29460800
C	1.32318800	1.47097600	0.87720100
S	0.27615900	-0.60416900	-0.20178400
C	-0.09532500	1.67574600	0.90644200
H	2.01420200	2.19438600	1.29316100
C	-0.80073800	0.63517500	0.35402200
H	-0.54407600	2.56219200	1.33666800
C	-2.23982300	0.47703900	0.19108400
C	-2.88501900	-0.68225100	0.65500200
C	-2.98400300	1.49303900	-0.43610800
C	-4.26323300	-0.82553100	0.49030800
H	-2.31586400	-1.46812300	1.15302600
C	-4.36062500	1.34808600	-0.60085700
H	-2.48225500	2.38996500	-0.79928200
C	-5.00176800	0.18770500	-0.13758900
H	-4.94788100	2.13179900	-1.08951400
H	-4.77380800	-1.72461900	0.84890800
C	-6.46775400	0.07211000	-0.33163600
O	-7.22239800	0.86731700	-0.84318900
O	-6.92164600	-1.12983200	0.17335700
C	-8.34531200	-1.39135000	0.04779200
H	-8.46303500	-2.32690100	0.60457700
H	-8.91752800	-0.57225200	0.49563400
H	-8.59890500	-1.50902900	-1.01038900

XYZ Coordinates of the lowest energy conformer of 4i

C	-7.75344000	-0.55599100	0.02078000
C	-7.31930000	0.59109500	0.71612800
N	-6.00448900	0.91350200	0.80001500
C	-5.11573500	0.07467800	0.17730400
C	-5.56050900	-1.08927500	-0.52071700
N	-6.87102900	-1.38953900	-0.59214400
H	-8.81180900	-0.82233700	-0.05306900
H	-8.01920300	1.26908400	1.21745700
H	-4.84819600	-1.77368600	-1.01435700
N	-3.76087600	0.47869400	0.28075400
H	-3.59806300	1.32484900	0.84035800
C	-2.65706600	-0.26166600	-0.21755600
O	-2.80243100	-1.35110900	-0.73864400
C	-1.35637700	0.39108300	-0.06531100
C	-0.98788700	1.69104500	0.17471800
S	0.03786500	-0.61684800	-0.26236600
C	0.43329200	1.86923400	0.21862400
H	-1.67104800	2.52034100	0.31220900
C	1.13010300	0.70487000	0.00986900
H	0.89147600	2.83131100	0.40756300
C	2.56606600	0.47564500	-0.02187800
C	3.15716700	-0.47838400	0.82123500
C	3.37223000	1.22101900	-0.90458600
C	4.53877100	-0.69106000	0.78338700
H	2.54361800	-1.05262500	1.51608000
C	4.74829800	1.00800600	-0.94628000
H	2.91403500	1.96088400	-1.56124800
C	5.32464500	0.04996400	-0.09907400
H	5.36484000	1.58570900	-1.63711700
H	4.97753800	-1.43332400	1.44776200
S	7.06609800	-0.10840800	-0.24512100
C	7.54894900	-1.46191000	0.84257300
H	7.07341300	-2.41047500	0.56763800
H	8.63660900	-1.58883200	0.73297400
H	7.34235200	-1.24663800	1.89703500

XYZ Coordinates of the lowest energy conformer of 4j

C	-7.21953900	0.28941700	0.14729800
C	-6.73913300	-0.55489800	-0.87533700
N	-5.41180200	-0.77050700	-1.04992600
C	-4.55780800	-0.13204700	-0.18751200
C	-5.04836400	0.72616500	0.84210700
N	-6.37142800	0.92516600	0.99826700
H	-8.28881900	0.46760000	0.29616400
H	-7.41176700	-1.07040800	-1.57037400
H	-4.36491400	1.25142300	1.53226000
N	-3.18554400	-0.41852100	-0.40802200
H	-2.99005100	-1.03274600	-1.20842800
C	-2.11523900	0.17707500	0.30378800
O	-2.29992800	1.03697200	1.14377800
C	-0.78396200	-0.33140200	-0.04179600
C	-0.35921700	-1.49037800	-0.64179800
S	0.55925300	0.65452000	0.41909500
C	1.06829300	-1.57237900	-0.74143500
H	-1.00400200	-2.28309900	-1.00177300
C	1.70649500	-0.47534900	-0.21653900
H	1.57124600	-2.41772100	-1.19446200
C	3.13512900	-0.19543500	-0.13276000
C	3.98521000	-1.12789200	0.48679600
C	3.65250700	0.99671800	-0.66594300
C	5.34965600	-0.83564600	0.55911400
H	3.58201600	-2.05006400	0.90586700
C	5.02738900	1.22714100	-0.56063500
H	3.00027600	1.71904700	-1.15835200
C	5.91523500	0.33510700	0.04641500
H	6.98378000	0.53999700	0.11633100
F	5.51832900	2.35608400	-1.06713400
F	6.15446200	-1.71855900	1.14649100

XYZ Coordinates of the lowest energy conformer of 4k

C	-7.35705400	0.01995400	-0.32814700
C	-6.78106200	-1.26728900	-0.32492100
N	-5.44109600	-1.44318900	-0.21230300
C	-4.67116400	-0.31368800	-0.10092900
C	-5.25863300	0.98753400	-0.11045300
N	-6.59220000	1.13887200	-0.22203300
H	-8.43701700	0.17066400	-0.41702600
H	-7.38560500	-2.17713000	-0.41332500
H	-4.64252700	1.90050100	-0.03180400
N	-3.28216600	-0.56581700	0.03645800
H	-3.01044500	-1.55605300	-0.00587800
C	-2.28314900	0.43830300	0.10080400
O	-2.55143400	1.61901300	-0.01256400
C	-0.92339100	-0.06072900	0.32151600
C	-0.44070200	-1.22327200	0.86879700
S	0.36983300	0.99692700	-0.12190100
C	0.98981400	-1.26463200	0.92043200
H	-1.04935300	-2.03646300	1.24632000
C	1.58156100	-0.13046900	0.41465600
H	1.52747500	-2.10903900	1.33393600
C	2.96400600	0.20567500	0.28508300
C	3.61838000	1.38248500	0.53255100
S	4.08894400	-1.01236900	-0.27947100
C	5.03362700	1.31938900	0.27237800
H	3.14961700	2.28851700	0.89940300
C	5.43559800	0.09369300	-0.16938600
H	5.68672900	2.17056500	0.42208300
Cl	6.99961300	-0.37572800	-0.57767000

XYZ Coordinates of the lowest energy conformer of 4l

C	-7.01179600	0.06400200	-0.20954600
C	-6.44640300	-1.22402000	-0.30405800
N	-5.10500100	-1.41541200	-0.24241700
C	-4.32257000	-0.30013900	-0.08324600
C	-4.90041000	1.00304300	0.00631400
N	-6.23491100	1.16953500	-0.05604800
H	-8.09236900	0.22693100	-0.25677700
H	-7.06040300	-2.12268700	-0.43137200
H	-4.27422300	1.90499600	0.12396800
N	-2.93304700	-0.56795100	-0.00353300
H	-2.66994300	-1.55500600	-0.11455200
C	-1.92299000	0.42466800	0.09652900
O	-2.18913100	1.61114600	0.06742700
C	-0.56322300	-0.09447800	0.24360400
C	-0.07047500	-1.30439700	0.66590100
S	0.72508100	0.99998800	-0.12290200
C	1.35956900	-1.35158800	0.68478800
H	-0.67299300	-2.15048400	0.97435000
C	1.94700000	-0.17453000	0.27983500
H	1.90207500	-2.23322700	1.00238900
C	3.32505600	0.17112400	0.15458600
C	3.95824000	1.37692700	0.28560100
S	4.49434700	-1.07659100	-0.23680500
C	5.38206700	1.30819700	0.07361700
H	3.47011400	2.31204200	0.53409600
C	5.83510000	0.05528700	-0.21794800
H	6.00539700	2.18919200	0.14781600
C	7.19313000	-0.42876500	-0.49673600
H	7.52278200	-1.18407600	0.23624300
H	7.27238300	-0.89116700	-1.49493200
H	7.93632300	0.38478900	-0.46680300

XYZ Coordinates of the lowest energy conformer of 4m

C	8.64659600	0.13220100	-0.37496600
C	8.18822100	0.14515200	0.95921800
N	6.86625000	0.08148600	1.25356300
C	5.99559200	0.00293300	0.19706600
C	6.46316400	-0.00364500	-1.15098800
N	7.78176800	0.05921100	-1.42070200
H	9.71173100	0.18155600	-0.62140400
H	8.87476200	0.20760700	1.81141900
H	5.76630300	-0.05519200	-2.00592700
N	4.62964900	-0.08644100	0.57429100
H	4.45146800	-0.03827000	1.58530400
C	3.54523900	-0.09818300	-0.33527400
O	3.70581100	0.02004900	-1.53489900
C	2.22427900	-0.27780300	0.27859900
C	1.82228000	-0.79276000	1.48431000
S	0.86282300	0.18208900	-0.68486900
C	0.39579100	-0.79841400	1.63789900
H	2.48131100	-1.17362200	2.25567100
C	-0.26161700	-0.28984400	0.54646600
H	-0.08961000	-1.16641000	2.53332600
C	-1.69266200	-0.13223800	0.32584400
C	-2.26522800	1.15068200	0.27743500
C	-2.49977600	-1.26881200	0.16435900
C	-3.64066300	1.28567100	0.06428800
H	-1.62356100	2.02694400	0.41347300
C	-3.87778000	-1.12063300	-0.04788600
H	-2.04388000	-2.26357700	0.20291500
C	-4.45371500	0.15308100	-0.10035200
H	-5.52920400	0.27673600	-0.26735900
C	-4.28930500	2.64459400	0.00761200
C	-4.71268400	-2.36341200	-0.21557700
F	-4.92075200	2.89734300	-1.15085700
F	-3.47371700	3.69868500	0.16257100
F	-5.23486800	2.83287500	0.94351000
F	-6.02703600	-2.17702800	-0.40769200
F	-4.65908300	-3.19614000	0.83805000
F	-4.34243700	-3.12915700	-1.25543900

XYZ Coordinates of the lowest energy conformer of 4n

C	-8.28880300	-0.08264400	0.32772600
C	-7.80689200	-0.60070700	-0.89260400
N	-6.47855500	-0.67410700	-1.15472200
C	-5.62513600	-0.22369400	-0.18037700
C	-6.11736100	0.30554800	1.05018000
N	-7.44152800	0.36814000	1.29011300
H	-9.35891500	-0.02139100	0.54810700
H	-8.47925700	-0.96376500	-1.67840200
H	-5.43526900	0.68042000	1.83342600
N	-4.24960000	-0.35256400	-0.50638700
H	-4.05601900	-0.72583100	-1.44403300
C	-3.18567300	0.11700900	0.30150500
O	-3.38143300	0.72370000	1.33746100
C	-1.84219500	-0.19798200	-0.19522600
C	-1.37566600	-1.10680000	-1.11106500
S	-0.53829100	0.68814800	0.51627000
C	0.05073300	-1.07295200	-1.25033900
H	-1.98847300	-1.79755200	-1.67805300
C	0.64742800	-0.13951400	-0.43852900
H	0.58033600	-1.71935500	-1.93916400
C	2.05850900	0.18612200	-0.29214200
C	2.49578000	1.51768800	-0.39122300
C	2.98778800	-0.84079500	-0.04913200
C	3.85638000	1.79125600	-0.24164700
H	1.78840400	2.32678700	-0.59068500
C	4.34761900	-0.53596300	0.09463800
H	2.63766500	-1.87339600	0.03276700
C	4.79762800	0.78582800	0.00135800
H	5.85367500	1.04563400	0.11265100
C	5.31045100	-1.66774300	0.35268300
F	6.60577400	-1.33638100	0.46413100
F	5.30587600	-2.61190000	-0.60434200
F	5.05766100	-2.34952400	1.48266700
Cl	4.39543600	3.41944000	-0.36215300
